# Technical and management coaching for government institutions: Lessons learned and health systems transformations across 8 countries in sub-Saharan Africa and India

**DOI:** 10.1371/journal.pgph.0004058

**Published:** 2025-01-03

**Authors:** Kate Graham, Anna Kalbarczyk, Lekan Ajijola, Emily Das, Fatimata Sow, Kenneth Owino, Maheen Malik

**Affiliations:** 1 Department of Population, William H. Gates Sr. Institute for Population and Reproductive Health, Family and Reproductive Health, Johns Hopkins Bloomberg School of Public Health, Baltimore, Maryland, United States of America; 2 Department of International Health, Johns Hopkins Bloomberg School of Public Health, Baltimore, Maryland, United States of America; 3 Johns Hopkins Center for Communications Programs (CCP Nigeria), Abuja, Nigeria; 4 Population Services International India (PSI-India), New Delhi, India; 5 IntraHealth International, Dakar, Senegal; 6 Jhpiego, Nairobi, Kenya; PLOS: Public Library of Science, UNITED STATES OF AMERICA

## Abstract

Traditional engagement with local governments often relies on financial and human resources from international or local partners, leading to direct implementation by organizations, which can hinder sustainability. While some organizations include sustainability indicators, few focus on transferring technical and financial ownership to governments. The Challenge Initiative (TCI) uses a phased coaching model—lead, assist, observe, and monitor—to build local government capacity for scaling family planning (FP) and adolescent and youth sexual and reproductive health (AYSRH) programs. TCI ensures local governments take the lead from day one, coaching them to manage and implement these programs sustainably, improving management, coordination, planning, budgeting, and data-driven decision-making. In-depth interviews (IDIs) and focus group discussions (FGDs) were conducted with stakeholders across 24 TCI sites in 8 countries—Benin, Kenya, India, Niger, Nigeria, Senegal, Tanzania, and Uganda—from October 2020 to March 2021. A total of 175 IDIs and 14 FGDs were conducted with 343 master coaches (52 TCI staff, 164 local government staff) and 127 coachees. Participants included TCI staff, local government officials, and health providers at both facility and community levels, all of whom received coaching. Examples were gathered to assess the impact of TCI’s coaching model on government FP and AYSRH programs. TCI documented improvements in local government and health service provider leadership, management, and coordination of health programs through its coaching interventions. It linked health system outcomes to its coaching approach, building a cadre of master coaches and coachees at all levels. This strengthened local governments’ capacity to lead and sustain the scale-up of FP and AYSRH programs beyond TCI’s direct support. Improved monitoring and tracking of coaching quality is needed for further insights.

## Introduction

In global health and development, traditional engagement with local governments has focused on “leading by doing” [1]. As a result, organizations directly implement programmatic activities on behalf of the government, hindering the government’s ability to deliver and sustain quality health programs and outcomes [2]. To address this challenge, recent efforts are focusing more on working collaboratively with governments to design and manage local initiatives. Transferring capacity to enable local governments to implement these initiatives takes many different forms, including mentorship, coaching, training, and supportive supervision [3–5]. While coaching is more often associated with the business sector through executive coaching models, the principles are becoming more widely accepted in the health sector [6]. The literature describes coaching as “systematically increasing the capability and work performance of someone by exposing him or her to work-based tasks or experiences that will provide the relevant learning opportunities and giving guidance and feedback to him or her to learn from them” [7].

Effective coaching strengthens coaches’ skills and ability to complete their jobs in the face of challenges and increases their overall leadership capabilities. Previous research has identified numerous benefits, such as improving performance, self-efficacy, motivation, and sustainability, [8–10] however, a gap still exists in assessing the adaptation of coaching to meet the stakeholders’ specific needs at various levels of the health system and the effects of coaching on motivation and quality of care [11].

Through this research, we aimed to reveal the government’s perception of TCI’s coaching and its effectiveness towards strengthening their capacity to lead and drive sustainable program implementation.

### TCI’s approach to coaching

The Challenge Initiative (TCI)–led by the William H. Gates Sr. Institute for Population and Reproductive Health at the Johns Hopkins Bloomberg School of Public Health–is a demand-driven approach that supports local governments in rapidly and sustainably scaling family planning (FP) and adolescent and youth sexual and reproductive health (AYSRH) global high-impact practices [12] and other interventions (HIPs & HIIs) [13] for the urban poor. In an ever-evolving global health field, leaders need to be able to make fast-paced decisions and respond to changing conditions, as was witnessed with the COVID-19 pandemic. While traditional command and control leadership and management styles provide a structure for managing incidents, it was noted that a creative, innovative and flexible approach to management is essential to finding solutions and better communication [14] to continue to deliver quality health services in the face of global pandemics, commodity stock-outs and other challenging circumstances as they arise. Therefore, TCI recognized that the development landscape required new thinking and a novel approach to support local governments and civil society organizations to become more efficient and effective managers, leaders and coaches.

As a result, TCI created the **Lead, Assist, Observe, Monitor (LAOM) coaching model**–defined as a structured yet flexible process by which coachees are empowered to make positive changes in their internal motivation, knowledge, skills and ability to address needs, solve problems, take on new challenges, improve individual performance, achieve individual team and organizational objectives and coach others in their geographies. This unique and systemic approach to coaching is locally led, owned and implemented and was modeled from Malcolm Knowles’ Adult Learning Theory (Andragogy) which considers coaching as the “art and science of helping adults learn” [15]. For the purposes of this manuscript, we define a **master coach** as the individual responsible for leading the cascade training of coachees, ensuring institutionalization of HIPs & HIIs for long-term sustainability. Ulrich defines a master coach as “developing both a philosophy and process that will be uniquely tailored to you as a coach and to the person you are coaching” [16]. A master coach may be a TCI hub staff member, a trained government staff member (from state, district, medical region, city, local government area, national, county or country level) or a lecturer or teacher at a health institution. A **coachee** on the other hand, is the recipient of coaching from a cascade training led by a master coach. Typically, a coachee has direct contact with potential and existing FP clients, including health service providers, which includes facility-based staff such as physicians, nurses, or pharmacists, or community-based health workers such as social mobilizers, or municipality staff and more. The relationship between coaches and coachees is critical but more research is needed to closely examine this relationship for its effectiveness in achieving outcomes [6].

**Fig 1:**
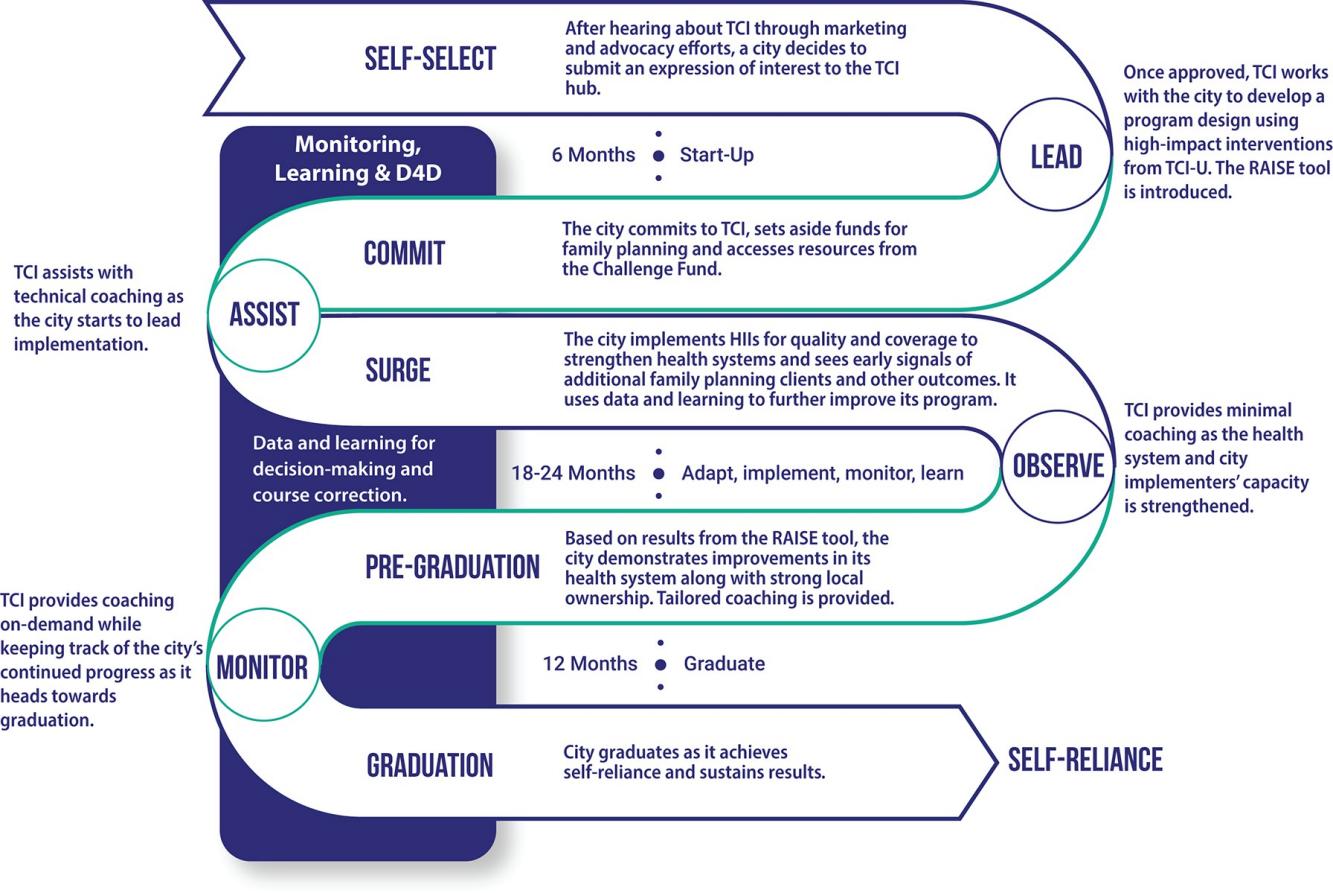
TCI’s lead, assist, observe and monitor (LAOM) model of coaching.

TCI’s engagement with a local government follows 4 distinct phases over the course of a 3-to-3.5-year period: 1) start up, 2) surge (implementation), 3) pre-graduation, 4) graduation and beyond (Fig 1) with coaching intensity declining as local governments move along the LAOM continuum.

The LAOM designations, however, do not squarely fit into a specific phase at one given time. Coaching typically starts out at high intensity (Lead) in new locations, where TCI staff provide technical (HIP & HII intervention-focused) and management (soft skills such as program management, coordination, leadership, planning, budgeting, and use of data to inform decisions) coaching to government staff during initial start-up and demonstrate how best to scale-up and implement HIPs & HIIs. Local government counterparts soon start to own implementation and TCI staff scale back their coaching to ‘Assist’ when requested by the government or when a gap is identified. The goal during direct engagement is for TCI staff to take on the ‘Observe’ role–that is, to serve solely in an observational or supportive role as local governments lead all management and technical coaching, ensuring sustainability of implementation of HIPs & HIIs beyond TCI’s support. From the outset, TCI’s approach to coaching sets local governments on a path towards self-reliance, and they eventually “graduate” from TCI’s direct support in around 3.5 years [17]. Coaching can then be requested on demand if needed, after they have graduated.

TCI’s coaching model uses a blended learning approach to meet the needs of hub city managers, local government and service providers and other stakeholders in partner geographies. This includes TCI University, a dynamic free online platform that offers FP and AYSRH HIP & HII interventions for learning, adapting, disseminating and coaching. As a key mechanism to build capacity and strengthen health systems, TCI-U supports local governments to scale up these interventions with in-person and virtual coaching, online toolkits and a vibrant community of practice. By coaching those in the public health sector, TCI seeks to put local governments in the driver’s seat from day one to help reduce turnover as local governments see a system that invests in them, ensure solid leadership succession by transmitting values and behaviors to the next generation of leaders, improve performance and productivity of both a coach and coachee, create a supportive working culture and create trust and build better professional relationships. Through this approach, many master coaches and coachees are created at each level of the local government health system to ensure continued quality implementation and impact beyond the period of engagement with TCI.

From 2016–2021, TCI’s hubs–Jhpiego in East Africa, IntraHealth in Francophone West Africa, Johns Hopkins Center for Communication Programs (CCP) in Nigeria, and Population Services International India (PSI-India)–embedded coaching systems within their local structures to facilitate and produce the rapid, cost-efficient expansion of accessible, quality urban FP and AYSRH HIPs & HIIs, led and sustained by 95 local governments across the 4 hubs.

## Methods and materials

### Study design

This multi-country, cross-sectional qualitative case study sought to identify in-depth documentation of TCI’s coaching model, compare its impact across locations, and derive best practices to inform current and future engagement with local governments. This study was conducted from October 2020 to March 2021 across 8 TCI project countries: Benin, Kenya, India, Niger, Nigeria, Senegal, Tanzania, and Uganda. Focus groups (FGD) and in-depth interviews (IDIs) were conducted for primary data collection.

### Participant sampling and recruitment

TCI engaged 3 main groups of study participants: **(1) TCI staff** (who are considered *master coaches* and who are responsible for coaching local government staff), **(2) local government staff** (who are considered *master coaches* and who have received coaching from TCI and also provide coaching to other government staff or to service delivery providers), and **(3) health service delivery providers** who implement FP and AYSRH HIPs & HIIs in their locations (who are considered *coachees* and who receive coaching from local government staff as well as TCI staff). All participants were recruited on a voluntary basis and were informed about their right to refuse or withdraw from the study at any time. Participants were also informed about the purpose, process, and benefits of the study. TCI selected participants based on a set of inclusion and exclusion criteria and then conducted purposive samples of TCI staff, local government staff and health service providers they support. In total, 343 men and women aged 18 and older participated in the study (Table 1).

**Table 1:** Participants by TCI hub.

*Participant Category*	*No. Interviewed*
*TCI Staff Master Coaches (includes Chief of Party*, *city/county managers*, *M&E manager*, *technical teams)*	11 (Nigeria)
	9 (India)
	10 (FWA)
	22 (EA: UG, TZ, KE)
*Local Government Master Coaches (includes district, medical regional officials, officials from relevant government health ministries, departments and agencies, country health providers)*	31 (Nigeria)
	17 (India)
	62 (FWA)
	54 (EA: UG, TZ, KE)
***Total Master Coaches***	**216**
*Service Provider Coachees (includes facility and community service providers including doctors, nurses, pharmacists, social mobilisers, municipality teams)*	24 (Nigeria)
	27 (India)
	28 (FWA)
	48 (EA: UG, TZ, KE)
***Total Coachees***	**127**

### Inclusion and exclusion criteria

TCI staff selected to join this study have worked with TCI for at least 6 months and provided substantial coaching support to local governments, while local government staff selected to participate have engaged with TCI for at least 2 years and have received coaching from TCI as well as have coached other colleagues and service providers. Service providers included in this study have been coached by TCI or the local government on the HIPs & HIIs and have implemented the interventions as well. TCI locations (city, state, county) were included in the study if they had engaged with TCI for at least 2 years and had a large population. TCI locations experiencing conflict, political unrest or turnover or that were difficult to access were excluded.

### Data collection

TCI developed a structured in-depth interview (IDI) guide that was used across country settings. The study teams in-country pilot-tested, conducted mock interviews, and refined guides based on the results. In some countries, a focus group discussion (FGD) guide was developed to help generate shared opinions on set questions and was based off the IDI. Guides were then translated into Swahili, Hindi, and French, as appropriate. Verbal consent was received, and witnessed by the study team, from all participants prior to interviews taking place. The interviews were administered by the TCI study teams, typically involving a 2-3-person team, and explored the experiences, perspectives, benefits, challenges, recommendations and sustainability of TCI’s coaching. Because this study took place during the COVID-19 pandemic, most interviews were conducted virtually on Microsoft Teams or Zoom and lasted around 60–90 minutes. Focus group discussions were used to delve deeper and extract and examine specific narratives from a group of participants, including community health workers, service providers and local government staff, on TCI’s coaching. Focus group discussions often took place in-person, however in some instances, they were also conducted virtually. Nearly all IDIs and some FGDs were recorded, however, if a participant did not consent to an audio recording of their interview, detailed notes were used to capture conversations for inclusion in the study. All interviews and FGDs were transcribed and translated into English.

A total of 175 in-depth interviews and 14 focus group discussions (FGDs; n = 168) were conducted across 8 countries located within TCI’s 4 hubs: East Africa, Francophone West Africa (FWA), India, and Nigeria. In India, 50 IDIs and 3 FGDs (n = 3) were conducted across 5 cities and 3 states; in Nigeria, 60 IDIs and 3 FGDs (n = 6) were implemented across 5 states, in Francophone West Africa, 65 IDIs and 5 FGDs (n = 35) across 5 cities took place and in East Africa, 3 FGDs (n = 124) were conducted across 9 cities and 3 countries (Table 2).

**Table 2:** The number of participating sites, IDIs and FGDs by hub.

*TCI HUB*	*COUNTRY*, *STATE*		*TCI STAFF*	*LG*	*HEALTH SERVICE PROVIDER*	*TOTAL*	*FGD*	*IDI*
	*CITY*, *COUNTY*							
	*(NO*. *OF INTERVIEW SITES)*							
***EAST AFRICA***	*Kenya*	*Migori*, *Kilifi*, *Nairobi*	*22*	*54*	*48*	*124*	*3 (n = 124)*	*0*
	*Uganda*	*Buikwe*, *Kiira*, *Kampala*						
	*Tanzania*	*Ilala*, *Ubungo*, *Arusha City*						
***FRANCOPHONE WEST AFRICA***	*Benin*	*Ucoz*, *Abomey-Calavi*	*10*	*27*	*28*	*65*	*5 (n = 35)*	*65*
	*Niger*	*Niamey*						
	*Senegal*	*Nioro*, *Ziguinchor*						
***INDIA***	*UP*	*Varanasi*, *Agra*, *Mathura*	*9*	*17*	*27*	*53*	*3 (n = 3)*	*50*
	*MP*	*Indore*						
	*Odisha*	*Berhampur*						
***NIGERIA***	*Rivers*		*11*	*31*	*24*	*66*	*3 (n = 6)*	*60*
	*Niger*							
	*Plateau*							
	*Ogun*							
	*Taraba*							
**TOTAL**	*24*		*52*	*129*	*127*	*343*	*14 (n = 168)*	*175*

### Qualitative data analysis

Interview transcripts were analyzed using thematic content analysis. This approach is used to identify, organize, and record patterns in rich detail in qualitative datasets. All IDIs and FGDs were coded for themes that emerged from the data. Codes and sub-codes were developed with reference to the interview guide and transcripts and the themes were later organized with the aim of connecting patterns and data relationships. Quotes, stories, and observations served as analytic documentation and were used to corroborate thematic summaries. Analysis was conducted in Dedoose, NVivo, and Microsoft Excel.

### Ethical approval

The Johns Hopkins Bloomberg School of Public Health (BSPH) Institutional Review Board reviewed the study protocol and deemed it non-human subject’s research.

### Inclusivity in global research

Additional information regarding the ethical, cultural, and scientific considerations specific to inclusivity in global research is included in the (**S6 File**).

## Results

Key themes from across 4 hub coaching studies were consolidated, analyzed, findings compared, trends identified, and key learnings and best practices were derived for TCI’s coaching approach. The study revealed 4 broad themes: coaching frequency and intensity, usefulness and benefits, challenges and improvements and sustainability of TCI’s coaching model.

### Coaching frequency and intensity

#### Coaching approach

After transferring the complete package of coaching to TCI hub partners, coaching plans were developed and coaching sessions were conducted at all levels of the local government system, from the state and national level down to the service provider level with the goal of empowering government officials to make positive changes in their internal motivation, knowledge, skills, and ability to address needs and solve problems within their FP and AYSRH programs. On average, each city had approximately 10 master coaches, and each held roughly 8 sessions in their monthly coaching plans. TCI staff collected and reviewed these plans to provide more targeted coaching support and feedback to the local government during supportive supervision visits.

#### Frequency

The frequency of coaching sessions varied depending on the phase of engagement the city was in (i.e., from start-up to graduation). On average, a master coach held between 2 and 15 sessions per month, with India and Nigeria noting the highest average, between 10 to 12 sessions.

To ensure coaches had a strong handle on both the management and technical coaching skills (i.e. improving management and coordination abilities, using data to inform local government problem-solving and decision-making, and technical coaching on FP and AYSRH HIPs & HIIs using TCI-U), it was noted that on average, it typically took between 3 to 12 months to move from Lead to Observe phase, with the average being 6 months; however, it depended on an individual’s skills, availability and implementation processes or approaches that were used. Overall, the coaching frequency decreased as the skills and confidence of the coaches were built and strengthened over time.

*In India*, *when speaking about coachees and specifically community health workers like the Accredited Social Health Activists*, *also known as ASHAs*, *it can take anywhere between “12–18 months–it takes longer because they are also working on other [health] areas and there are different learning levels”*. *It was noted that for coaches*, *it can take “within 3 months to assist*, *and 6 or more months to move into observe stage*.*” India*, *IDI*, *Coach*

*In Plateau State*, *TCI coaches suggested that from Assist stage to the Observe stage could be 3 to 6 months*, *but it is dependent on the individual’s availability and implementation processes or approaches that are to be used*. *“But no matter what*, *I think that within 3 months to 6 months should be good*, *If on the average*, *you interact with the person*, *on the average of 2 hours a week*, *or 1 hour per week*, *and the intervention in question is a simple one to carry out*, *for example*, *whole site orientation*, *QIT [Quality Improvement Team]*, *that one you will see that Niger state people have started running with that one because it’s easier*.*” Nigeria*, *IDI*, *Coach*

#### Method of knowledge transfer

Two modes of knowledge transfer were identified from the study, face-to-face sessions, and virtual sessions. Face-to-face meetings were the most requested medium for coaching through on-the-job coaching, class or hall settings, supportive supervision visits, observations, group discussions, practical sessions, and neighborhood campaigns. This allowed for a more supportive supervision approach where coaches could observe teams performing various activities to identify capacity gaps. For instance, in cases where the coach felt the topic was minor, it could be addressed via a phone call. However, in cases where addressing the capacity gap needed the involvement of several people or practical sessions, then face-to-face coaching was preferred.

When coaching transitioned to a virtual platform during the COVID-19 pandemic, some coaches reported that they lacked confidence in handling a digital platform and it took time to adapt to new technology. Internet connectivity also posed a challenge, and the quality of interactions, fluency and regularity of touch points were sporadic. However, after some coaching on the use of technology, the coaches began to adopt digital means of connecting with their coachees, TCI and their fellow colleagues and activities and regular coordination continued virtually through Zoom and WhatsApp.

Coaches expressed the need to appreciate individual differences, which would also influence the best method of coaching. While some individuals were fast learners and comfortable with the virtual mode, others had technological challenges which made virtual coaching impossible. Overall, coaching was noted as being highly adaptable and after COVID-19 restrictions were lifted, government staff became more open to virtual coaching, which has remained in place through WhatsApp Groups.

*“There is no one shoe that fits all when it comes [to]…the delivery method to coach*. *First*, *I have to consider the time available for both coach and coachee*. *Second*, *I also look at their capacity*, *some people are tech- savvy and are able to understand things even when you teach them virtually or even on phone*. *Lastly*, *I consider if it is a follow-up and then choose the best*.*” East Africa*,* FGD*, *Coach*

### Usefulness and benefits

#### Positive changes

All study participants reported that TCI’s coaching approach empowered TCI hub staff, local government officials and service providers to make positive changes. Such positive changes would include increasing confidence, knowledge and skills, and the ability to address pressing needs, solve problems, take on new challenges, improve individual performance, and achieve individual, team, and organizational objectives. The trained coaches cited examples of improvements in their problem-solving skills and mindsets at all levels, starting from the state to the city and community levels.

*“The more you coach*, *the more skills you get and that’s a change*, *because if you just sit on the knowledge*, *you don’t advance*, *but as you keep on sharing*, *coaching others you end up like becoming perfect and you gain skills*.*” East Africa*, *IDI*, *Coach*

*“They [coachees] have developed a confidence level and interest towards their work and this will keep them moving without any problem*.*” India*, *IDI*, *Coach*

*“As a coach*, *TCI’s coaching approach is a strategic shift in mindset towards facilitating building skills and particularly in upgrading knowledge of our health provider peers” Francophone West Africa*, *IDI*, *Coach*

In India, the coaching approach helped build good relationships between the coach and coachees. Participants who underwent coaching identified trust, honesty, transparency, active listening, spontaneity, knowledge, and skill, and improvement in their ability to coordinate as critical attributes that make coaching effective. During coaching sessions, it was essential to create a positive atmosphere and show appreciation for both the coach and the coachees. Because of TCI’s coaching, government stakeholders said their confidence and skills to advocate and present data in the state level platforms as well as at all levels of the health system were greatly improved.

*“Since I have been trained [coached] so well at this time*, *if anyone talks with me about FP*, *I won’t hesitate*. *Instead*, *I… want to talk about FP because I am aware now*.*” India*, *IDI*, *Coachee*

In Nigeria, local government coaches reported that they are now more conversant and confident with skills in interpersonal communication, program management, as well as FP and AYSRH program coordination and implementation. The government coaches and service providers agreed that the support they received from TCI changed their coaching strategies and helped build their capacity in coaching and service delivery, as well as public speaking skills. In addition, they acknowledged that they had more patience, resilience, and ability to carry out various tasks in a structured and logical manner.

“*Through TCI*, *I’ve been able to put this training that I have received as well into practice*. *During the cause of my interaction with the community and the mobilizer as well*, *I have been able to impact most of this knowledge into the community as well so by and large TCI has helped in a great way to improve my own knowledge and at the same time improve FP information and uptake…*” *Nigeria*, *IDI*, *Coach*

*“…sometimes I used to have fear; my heart used to beat*. *But due to the coaching that I’ve been receiving*, *I have more self-confidence and I’ve improved in many aspects of my job*, *including inserting implants and counseling…” Nigeria*, *IDI*, *Coachee*

In East Africa, better engagement and commitment of geography teams, better inter-sectoral and interdepartmental collaboration, and a better understanding of the engagement timeline were perceived as positive changes because of coaching. TCI coaches seemed to agree that continued coaching brought noticeable changes in attitudes and subsequent ownership of FP and AYSRH HIPs & HIIs.

*“I think in some of the geographies*, *the commitments that the geographies have made*, *this is like the first the commitments are being made and that has improved or provided the basis for continued investment by these geographies in FP and AYs*. *I think that TCI has contributed to that aspect of health in terms of providing an avenue in which the county can continue to invest resources for RH*, *FP and AY*.*” East Africa*, *IDI*, *Coach*

Through the coaching of mayors in FWA, many now understood the benefits of FP for the population and were now FP and AYSRH champions in the community and helped mobilize financial resources for FP and AYSRH programs. Coaching is now standardized, and coaches come prepared with a coaching plan contextualized for the area of coaching needed. In addition, due to TCI coaching, the hub and regional staff were more accountable and performed more effectively to secure city-level impact.

*“I can see now that they [local government] understand the TCI model and don’t wait for TCI to do everything*. *With the managerial coaching municipalities have been receiving*, *they now participate directly in improving the quality of FP services provided in healthcare facilities like for example through the hiring by the mayor of 2 female midwives for the TCI program in 2 of TCI FWA implementing cities*. *They are implementing and are seeing their performance improve*, *and everyone is executing against the milestones” Francophone West Africa*, *IDI*, *Coach*

#### Coordination

Participants noted that specific structures existed in cities prior to TCI engagement, for example, city coordination committees in India and program implementation teams in East Africa; however, these were generally either non-functional or organized on an ad hoc basis with little to no follow up. TCI’s coaching emphasized the importance of these groups having regular touch points to track outcomes and bridge gaps. In addition, TCI’s coaching supported the operationalization of these mechanisms across all hubs which ensured coordination between departments and clarity in roles. These coordination meetings also ensured cross-departmental coaching, learning, understanding of each other’s work and promoted the use of data for decision-making. As a result, there were fewer coordination challenges than there used to be and a clear delineation of roles and responsibilities to ensure accountability in all aspects of programming.

“*TCI’s initial coaching approach focused mainly on strengthening the capacity of coaches to scale up TCI interventions*. *However*, *the coaching support received by these government officials now includes strengthening leadership skills*, *program management*, *coordination*, *planning*, *budgeting*, *and use of data to informed decisions to ensure the sustainability of implementation beyond TCI’s support*.*” Nigeria*, *IDI*, *Coach*.

Finally, because of engagement with TCI, several participants reported positive changes in different departments now working together to promote FP service delivery. For instance, the public and private health sectors are now reportedly working together, and various non-health sectors, such as education, also collaborated with the health sector during the regular coordination committee meetings.

#### Health systems strengthening

Most participants indicated that while coaching was focused on implementing technical interventions, such as the HIIs & HIPs, in the beginning, over time, more emphasis was paid to providing management coaching that helped strengthen the overall health system. It was noted that implementing the interventions alone would not create the lasting impact that the cities have seen, without being coupled with management coaching. Since 2017, nearly 11,000 local government coaches have been equipped with both technical and management skills; they are now providing health care in a more efficient manner, through knowledge sharing and skills-transfer, coupled with better documentation and quality data generation for more informed decision-making. Between 2022–2023, TCI coaches supported the capacity strengthening of more than 18,000 community health workers and service providers with the necessary skills and knowledge in implementing HIIs & HIPs. Nearly all study participants noted that strengthening the local government’s capacity positively impacted resource allocation from the governments, which led to FP and AYSRH HIPs & HIIs being incorporated into city work plans as well as budgeted in program implementation plans, costed implementation plans, and annual work and financial plans. Though results varied by hub, 75% of all TCI supported local governments incorporated relevant HIIs & HIPs into their policies and budgeted plans. However, TCI began tracking this data in 2023 so though there are positive signals of institutionalization, there is room for improvement. By the end of December 2023, 85% of the funds committed for FP programs was spent by local governments ($11 million out of $15 million committed funding) [18]. Government staff and service providers mentioned that they now prioritize FP and AYSRH services in terms of health governance through systematic trickle-down coaching. Planning and budgeting of HIPs & HIIs and coaching led to the creation of coaching champions in the system that was found to positively impact the ability of various government stakeholders to better manage their daily engagements. Coaching was also seen to have diffused beyond the TCI-supported locations, through deliberate efforts or organically when already coached individuals were transferred to other stations or pursued opportunities elsewhere.

*“There is a lot of ownership now of the results that we generate and then there is an increase in quality in terms of data as opposed to earlier where data quality was low*, *there were many gray areas*. *So*, *with the adoption of the TCI model of doing things*, *there has been a change in terms of the services delivery within the geography*.*” East Africa*, *FGD*, *Coach*.

*“I have been working in the public sector since 2013*, *earlier I served many development organizations*. *Based on my previous experience I can say most development agencies bring their own resources to drive change*, *on the contrary*, *TCI India since its initiation utilized local government resources by involving city officials to cultivate change by creating ownership of evidence-based practices and sustaining the efforts within the system. TCI focused on strengthening the capacities of government staff involved at each level of the system and empowered them to take the lead in executing at their level*.*” India*, *IDI*, *Coachee*.

*“My goal as a TCI coach is to be able to*, *we use this word “cloning”*, *it’s to be able to replicate the skill and capacity I have to be able to reproduce multiples of myself in that state and I feel that’s the more sustainable way to leave all this impact and everything we have*. *I think that is the best way to improve on the human resources of the state*, *when it comes to health sector so for me*, *my goal is to be able to replicate myself in the state*.*” Nigeria*, *IDI*, *Coach*

The positive effects of coaching were seen to have led to self-reliance and institutionalization within the health care system. Strengthening the capacity of coaches at all levels was cited as key to sustainability of the interventions and participants also noted that the skills built were transferrable and would benefit other health services beyond FP.

### Challenges and improvements

#### Changes in human resources

The turnover of staff and government leaders specifically caused a challenge in continuing cascade coaching. It can take 6 months to 1 year of coaching government staff, on a weekly and monthly basis, to reach the Observe phase, so a transfer of a new coach to a new city also meant the master coach had to start from scratch to build a new coach’s capacity.

*“We are seeing total changes in the government structure*, *or the reallocation of manpower from one place to another or within the geography or outside the geography*. *So*, *when this happens*, *if you engage this person from the time*, *you are starting the project and she was on top of things*, *once she or he is reallocated to a different geography which is a non-TCI supported geography*, *obviously we can get some gaps on how things are working*, *comparing on how that person was in charge of things*.*” East Africa*, *FGD*, *Coach*

*“Attrition rates are more in government systems; until and unless my leadership is not changing then things are easier for the city…unlike changing of leadership which makes the things difficult because again transferring the vision and skills which acts as a barrier at medical officer in charge [MOIC] level*.*” India*, *IDI*, *Coach*

Although high turnover was presented as a major challenge by all hubs, it was noted that the institutional knowledge and skills were often left with those who moved on. However, the challenge remains that there is no tracking mechanism for these skills or individuals once they are transferred. Seeking coaches who hold long-term positions was noted as being critical in limiting high turnover rates.

*“Different levels of leadership*, *one level of leadership is always transferred (general manager at state level and chief medical officers, they get transferred every year)*. *Each city should have a plan for a master coach system in place so there needs to be a mechanism to track this*, *[and to] empower those that are around longer*.*” India*, *IDI*, *Coach*

*“TCI should look into [coaching] more people*, *so that the current coaches have good and competent successors to fill the gaps that could occur when the coaches and other trained personnel relocate or move to other opportunities as the tide changes*.*” Nigeria*, *IDI*, *Coach*

Though vacant positions exist due to this turnover, TCI’s coaching strategy ensures that capacity built at the local government level was transferred from one to coach another. In adult learning, peer-to-peer learning is generally culturally preferred, acceptable, and creates efficiency. This inter-geography learning has been a more acceptable way of showing new local governments that whatever they are being coached on has been done in another place with similar cultural context and has worked. This had helped overcome the challenge of lack of human resources at city level or until enough master coaches are created within the government system.

*“TCI should spread its tentacles to all the local government as to ensure the knowledge and know-how about the high-impact interventions gets to the local government as directly as well as train more mobilizers for greater population coverage*.*” Nigeria*, *IDI*, *Coach*

#### Internet connectivity and online resources

TCI coaches explained that TCI University is used as a reference to access toolkits and resources, and to learn and earn certifications and while coachees would occasionally register, it was not always a commonly used platform. This was attributed to a lack of computers, mobile devices, or internet access. This resulted in some government teams relying heavily on TCI staff for information rather than accessing these resources on the platform independently.

*“We need to ensure that all the tools that we are using for reporting and also for quality checklist are available there for ease of reference*, *because you will find sometimes someone is asking you for something and maybe yourself*, *you are not able to send*, *maybe you don’t have*, *maybe where you are*, *you are having problem with the internet or you are not able to login. And if you empower them*, *they have already been empowered*, *they know where to fine the tools we would rather have all the tools there so that at least it gets easy for them to be able to access them whenever they want them*.*” East Africa*, *FGD*, *Coach*

Difficulty accessing resources on coaching due to internet connectivity or lack of mobile device data persisted in most hubs; however, using printed versions of training aids and materials helped to mitigate some of these problems. Therefore, coaches mentioned that TCI should offer more offline learning materials to ensure coaches can always access all materials.

*“I find printed material most useful because on mobile phone only we can see this but printed materials can also be seen by the beneficiaries and we can take them to field as well for reference*.*” India*, *IDI*, *Coachee*

*“Let the learning materials be off-line so that more providers can have access*. *You know the data issues–there is no data*.*” Nigeria*, *IDI*, *Coach*

#### Time constraints and competing priorities

Making time for coaching and learning was another common challenge. Local government and service providers already had overburdened workloads and tasks that might be deemed more important. Irregularity in meetings could lead to gaps and inconsistencies in cascaded training. For example, coordination committee meetings did not occur routinely in every city due to busy schedules or other emerging priorities.

*“Despite balancing the coaching and other responsibilities*, *there are situations when the coaching sessions could not be done as per planned schedule*, *and the rescheduling of the sessions*, *intentional as well as on-demand*, *has to be done. Their engagements in other national programs forced them to dedicate major proportion of their time on the assigned job responsibilities*.*” India*, *IDI*, *Coach*

Participants reflected that there seemed to be no standard or prescribed means of identifying capacity needs or prioritizing them when it came to coaching. As a result, coaching would take time and require the coachees to be receptive and available to learning new things as well as their supervisor’s willingness to approve their time away.

*“When we listen*, *we are able to know any other key area they are struggling on. I can give an example that we may want them to go and learn from TCI University*, *but they don’t have the data*, *maybe they don’t have phones they don’t have time to read*, *like I give example the geography I support. So*, *at the end of the day*, *listening to them helps you to know that*, *okay*, *apart from this challenge is there anything that you can do to enable these people to have the learning*.*” East Africa*, *FGD*, *Coach*

During the COVID-19 pandemic, coaching shifted to virtual platforms, which proved time-intensive and a challenge for those not comfortable with technology. It was clear that face-to-face coaching was preferred, but participants cited time constraints as a challenge that often-necessitated virtual coaching.

*“In the Covid time we are facing challenge. People have the habit of physical meetings*, *which is not possible*, *therefore the city has moved towards the virtual meetings i.e.*, *how CCC [city coordination committee] has been done by CMO [chief medical officer] virtually. Therefore adapting the digital platform in a traditional way is becoming a challenge for us*.*” India*, *IDI*, *Coach*

Many coaches and coachees across regions emphasized the need to establish clear timelines and goals that are well understood and agreed upon from the outset. Given that TCI’s direct engagement is limited to about 3 years, clear expectations from the start are essential so graduation does not come as a surprise to local governments. This would also help the local teams appreciate that they have limited time to make best use of the coaching support provided.

*“I think it’s good to have a very clear…timeline so that you can work within it. Also, setting steps around how to carry out the coaching and I think there would be expectations by defining how the process should happen I think is also critical” East Africa*, *IDI*, *Coach*

#### Limited monitoring and better documentation

There was a need for greater documentation and tracking of coaching frequency, type, and related information for coaching (e.g., was it requested, subject matter, frequently asked questions and follow up). The hubs also noted the need to learn more about what kind of coaching is needed most at each step of the LAOM model to better plan and improve their coaching.

*“The data collection tools are hard copies making this time consuming and in some cases were not well understood (at first)*, *leading to many delays in reporting coaching activities. I think that it might therefore prove necessary to revise the reporting process to a much simpler one” Francophone West Africa*, *IDI*, *Coach*

*“Another gap that we have is in the documentation in the type of coaching because we document our coaching on ODK [open data kit] but sometimes we usually forget to record so that we are able to establish one pool of coaches and to the specific area which the coaching has been done*.*” East Africa*, *FGD*, *Coach*

Though many participants across the hubs mentioned that coaching led to better data quality and timely collection, participants noted a need to better learn how to analyze the data generated to inform decisions.

*“We need to coach them on data for decision-making*, *beginning even with us in terms of identifying the gaps. I attended another one recently with the PMA [Performance Monitoring for Action] data*, *and you find if you are not ready to coach them in that data for decision-making it’s back to business as usual. Maybe during feedback forums identify the gaps and see what to do with that data. What is the data telling us?” East Africa*, *FGD*, *Coach*

*“In managing the disparities between the technical and management personnel*, *data should be made available for those in the lower cadres (technical) to see the holistic picture of happenings at the technical level and summaries with insights at the managerial level*, *such as the Directors and the Permanent Secretaries*, *to facilitate better decision-making*.*” Nigeria*, *IDI*, *Coach*

Going forward, participants noted that the coaching model must address developing a proper monitoring and tracking system to ensure that the knowledge provided was not lost, given the frequent transfer of coaches. This system would ensure that the coaching model’s management and technical coaching aspects could be replicated in areas where TCI has not worked.

*“If coaching has been delivered*, *how do we ensure follow up of a coaching session in terms of outcomes and tracking. At the external level*, *[there should be] proper time and stability (coach someone on something and you go back and there is a new person in that position). Two to 3 chief medical officer changes happen in a few months (mid-sr. level positions often change). If a data operator leaves*, *a new person comes in. How do we strengthen these. Quality services*, *how to ensure this is happening*.*” India*, *IDI*, *Coach*

### Sustainability

Coaching and training sessions were instrumental in implementing the FP and AYSRH programs across the cities. To sustain TCI’s coaching model, participants pointed out the need for creating a cadre of master coaches in the system responsible for institutionalizing regular coaching. Identifying coaching champions at various positions in the public health systems who were least likely to be transferred would help retain coaching skills and coaching capacity and ensures the FP and AYSRH program continues to run smoothly. Participants also agreed that with continued coaching, there was a noticeable positive change in the attitude of geography teams with subsequent ownership of the project. Overall, building the technical capacity of the local government was cited as being key to the sustainability of the activities TCI initiated noted participants from across all hubs.

The sustainability of TCI’s coaching approach also depends on the cooperation and collaboration of local government stakeholders. Having strong government advocates and coaches that prioritize FP and AYSRH resource allocations, adopt and institutionalize HIIs & HIPs within costed implementation plans and government workplans, establish a culture of transfer knowledge through coaching of other government staff and service providers, host regular coordination meetings to plan interventions, fill gaps and monitor and assess the effectiveness of the interventions was critical to sustainability. It was noteworthy that in India, several aspects of the coaching model were already incorporated into the government program implementation plan, so there was a strong base for interventions to be institutionalized in the system due to set budgetary allocations. Participants noted that there are now budget allocations for training, service delivery and data-for-decision-making interventions as well as for the FP logistics management information system to ensure continuity of supply for FP methods. It was also noted that a budget has been set aside for regular coordination committee meetings across all hubs, to ensure that at the city level, FP data is regularly reviewed and discussed.

To measure progress towards sustainability, TCI developed the Reflection and Action to Improve Self- Reliance and Effectiveness (RAISE) assessment tool and supports local governments in implementing the tool quarterly [19, 20]. Through the coordination committee meetings, TCI and the local governments undertake RAISE assessments to regularly assess political and financial commitment, capacity strengthening, institutionalization and sustained demand of HIIs & HIPs. Many participants referred to the RAISE tool in their interviews because it provided a structured platform for a systematic review to assess progress, effectively conducted actions to bridge any gaps, and made course changes if concerns were detected throughout the evaluation process. The coaching plans were agreed to at the end of the quarterly assessment and, at the same time, evaluated coaching from the past quarter and agreed on plans for the next quarter.

## Discussion

TCI’s Lead, Assist, Observe, Monitor (LAOM) coaching model has been crucial for putting the local governments in the driver’s seat. Government stakeholders were involved from the beginning of the partnership with TCI and this collaborative process began with the codification and adoption of HIIs and HIPs, co-creation of the FP and AYSRH programs, cascading of LAOM coaching to a cadre of more government coaches within the city, and monitoring and tracking progress towards self-reliance and sustainability. As a result, at least 10 master coaches have been created within each city to help facilitate implementation of the HII and HIPs in expanding FP services to the urban poor. These coaches have further cascaded coaching and created a second line of coaches to not only sustain the momentum they’ve created but also sustain the impact they are seeing. In India, the coaching model was noted as a perfect blend of technical (focusing on HIIs & HIPs) and management (focusing on intersectoral coordination, capacity building of service providers, data, and commodities) components. It significantly transformed the way of working and the mindset change in the government stakeholders.

### Coaching empowers local governments

The TCI coaching model has been valuable in transferring skills to local government master coaches and, through cascade coaching, to various cadres of public health workers. TCI coaching model empowers and increases the engagement of local government stakeholders through technical and management coaching. It has also contributed to forming a community of practice network where coaches support each other, strengthening linkages between these program areas and ensuring a more coordinated approach to health service delivery. This has contributed to health systems-level changes to improve FP and AYSRH services. A growing body of evidence suggests that coaching improves the implementation of evidence-based practices, like TCI’s HIIs & HIPs, and is related to greater improvements in health outcomes [21]. It is also an important tool for sustaining practices over time. Redshaw argues that wherever possible, coaching should replace training to ensure long-term impact as, “coaching success depends on a lot more than just training” [7].

### No one-size-fits-all approach to coaching

Training a coach takes time and this varies widely by motivation, interest, and skillset. The International Coaching Federation, a membership organization for trained professional coaches, certifies different levels of coaches based on 60–200+ hours of coach-specific education in addition to 100–2500+ hours of client coaching experience [22]. And coach-specific education can vary widely, from short training courses to multi-month certification programs [22]. Learning how to coach is a complex process, and most coaching materials have been developed and studied in Western settings, [23] making tools and approaches possibly less applicable in different contexts. Within this study, it was noted that it took on average 6 months to coach an individual to become confident and comfortable on a topic, however this depended on the education, skillset, and interest of the coachee. Given these key considerations, there is no one size fits all approach to coaching.

### In-person coaching is preferred

Face-to-face coaching sessions were identified as the preferred method by both coaches and coachees. TCI’s coaching was also noted as being highly adaptable, especially during COVID-19 where coaching and supportive supervision were carried out over virtual platforms, the telephone, or WhatsApp. Post-COVID-19, TCI is seeing significant improvements in implementation with a combination of virtual coaching plus face-to-face coaching, as was noted by Artman-Meeker, who saw statistically significant improvements in workshop plus distance coaching versus those who participated in workshop only groups [24].

### Consider how coaches respond to requests

Coaching must also focus on the person being coached and not the coach themselves, as coachees are the ones that will make change happen on the ground [16]. However, there is increasing recognition that coaches need to become aware of and manage their own responses to questions, particularly related to diversity and inclusion (from culture, heritage tradition, faith, language, education, experience, gender, etc.), before they can help coachees to do the same [25, 26]. This has implications for the coach, how they are trained, and how they train others, and may be of particular importance to initiatives such as TCI, which seek to become more gender intentional in their programming and coaching.

### Coaching monitoring tools are needed

While coaching has been said to be valuable, coaches noted the difficulty in evaluating the effectiveness of coaching over time. Often coaches are unclear about how to measure success or report progress [27] and often coaches are evaluating impact on an individual or organizational level, as witnessed in this study. For example, in one country, success was measured based on supportive supervision reports and a coachees progress overtime, while in another country, better implementation of activities coupled with impact was seen as a marker for coaching success. De Meuse also noted the lack of research and literature on coaching effectiveness and a need to develop a more systematic approach to document and monitor coaching and the outcomes [28]. Based on a meta-analysis of coaching studies, [28] TCI plans to develop an appropriate instrument or tool to effectively measure coaching without coach/coachee rater bias issues. The tool will be in a standard questionnaire format, be appropriate for the type of coaching and level of coach and be specific to the objectives of the coaching engagement. This tool will be used to ensure proper follow-up on coaching sessions and allow coaches to better understand where improvement is needed and monitor coachees against performance goals.

### Coaching leads to sustainability

Family planning program implementers should prioritize coaching as a sustainable means to build capacity and reinforce best practices through continuous coaching efforts. Creating a cadre of master coaches, preferably those coaches who hold long-term positions, who are empowered to cascade technical and management coaching to other government coaches is critical to the sustainability of activities. Identifying coaches that feel a strong sense of ownership of their FP and AYSRH program, who will drive advocacy for adopting and implementing HIIs & HIPs, ring fence budget allocations for key interventions, and regularly assess their progress using data through coordination committee meetings has been shown to be critical aspects of sustainability. Other means of strengthening the capacity of local governments and service providers exist, however, whenever, and wherever possible, coaching should replace training to ensure long-term impact and more cost-efficient programming [7]. At all points of engagement, integrating a unified coaching plan with the local government team in all program activities is critical to sustainability.

### Strengths and limitations

This qualitative research captured both depth and breadth of experiences with coaching in a variety of international settings and with a large sample of participants. The documentation of this diversity of perspectives is an important addition to the coaching literature, which has long been developed for and studied in western settings. It is equally important to document coaching experiences across all countries, particularly in the resource-poor urban settings in which TCI operates. While we referenced the International Coaching Federation requirements for a trained professional coach, the number of hours referenced were from western studies. Participants from this study did note that the frequency of coaching sessions ranged from fortnightly to monthly and quarterly with each session lasting from 30 minutes to 2.5 hours, depending on the topic, and that on average, for a service provider coachee to feel confident implementing a specific HII & HIP, it took anywhere from 6 months to 1 year. Though TCI did not set standards in this same manner, what is important to note is that coaching is a complex process, and more information and monitoring is needed. In addition, TCI developed a standard IDI to generate evidence on personal opinions and following pilot testing of the guide, some study teams developed a modified guide for FGDs to help generate shared opinions, therefore there was some variation of the guides across the countries. Because this study was also conducted during the COVID-19 pandemic, some interviews were conducted in-person and some virtually, depending on the comfort of those being interviewed. Analyses conducted by TCI’s 4 hubs used different qualitative software tools with some teams using NVivo, Dedoose and one team using Excel. While these qualitative tools are similar, they also highlight limitations of TCI’s qualitative measurements and analysis. Despite this limitation, similar themes emerged across all hubs.

## Conclusion

The purpose of TCI’s coaching approach is to improve leadership abilities, program administration, coordination, planning, budgeting, and data utilization to aid in the sustainability of implementation after TCI engagement and direct support ends. Concrete examples illustrated how its coaching approach impacted local government and the health care system, empowered hub staff, local government officials and service providers to make positive changes. In essence, the coaching model has proven critical in energizing health systems and public health workers to provide FP services more efficiently and effectively. This model can also be adapted for coaching in other public health programs. Program managers and implementing partners should support local governments to institutionalize the coaching model for sustained provider capacity improvement.

The study highlighted several recommendations to improve coaching which included, strengthening the coordination bodies and the program coordination and feedback system, developing better tools to evaluate quality and improvement of coaching, rewarding, and recognizing master coaches and coachees who successfully lead and drive coaching sessions within their locations, and intensifying training to more master coaches and coachees.

## Supporting information

S1 File Gates institute in-depth interview guide questions. (DOCX)

S2 File East Africa focus group discussion guide. (DOCX)

S3 File Francophone West Africa focus group discussion & in-depth interview guide. (DOCX)

S4 File India focus group discussion & in-depth interview guide. (DOCX)

S5 File Nigeria focus group discussion & in-depth interview guide. (DOCX)

S6 File Inclusivity in global research questionnaire. (DOCX)
