# Supplementary material for: Technical and management coaching for government institutions: Lessons learned and health systems transformations across 8 countries in sub-Saharan Africa and India
Source: PLOS Glob Public Health. 2025 Jan 3;5(1):e0004058. doi: 10.1371/journal.pgph.0004058 (PMC11698439; doi:10.1371/journal.pgph.0004058)
Supplement: S1 File — (DOCX) [file pgph.0004058.s001.docx]

**Study Design and Questions**

| TCI Accelerator Hub Field Staff | Government (state, county, city) Officials parterning with TCI | Health Service Providers implementing TCI approaches |
| --- | --- | --- |
| **Coaching History & Support**   1. Have you attended any session/ training on coaching methodologies in the past?  - If yes, who conducted the training? - If no, do you feel a need to attend such a course?  1. How confident are you coaching (on a scale of 1-10)? What would make you feel more confident in your coaching ability? 2. [For City Manager] Do you receive guidance from Hub on how to coach? If yes, how often?  - Weekly - Monthly - Quarterly  1. [For Hub] What is your experience of coaching you receive from GI/ Global TCI team?  - Which aspects of your work has this coaching improved? - Is it coaching on HIA, management, data analysis or other technical areas, please explain? - Do you feel you need more support from Global? If so, what type of support? - What cross hub learnings, if any, have changed the way you coach or work in general? - In what ways, if any, has Global helped in your professional development? - Does Global add value to technical areas of your work? If it does, can you please provide an example?  1. What do you think is needed to build a good relationship between a coach and coachee? What reactions/responses do you receive from those you coach? | **Coaching History & Support**   1. Have you attended any session/ training on coaching methodologies in the past?  - If yes, who conducted the training? - If no, do you feel a need to attend such a course?  1. How confident are you coaching (on a scale of 1-10)? What would make you feel more confident in your coaching ability? 2. How frequently do you request coaching from TCI?  - Weekly - Monthly - Quarterly  1. What do you think is needed to build a good relationship between a coach and coachee? What reactions/responses do you receive from those you coach? | **Coaching History & Support**   1. Have you been coached on any family planning interventions? Probe: If yes, what interventions have you been coached on? 2. Did you find the coaching useful? Probe: what was useful? What wasn’t? 3. Are you aware of TCI University? Did your coach refer you to TCI University? 4. Have you ever requested coaching? And, to whom have you requested it from? Probe: How long did it take for the coach to respond to your request? 5. What has changed in your routine tasks since you received coaching? |
| **Current Coaching Experience**   1. Since you were hired to today, what areas do you coach on?  - Technical / (HIA) - Management - Others  1. Who do you coach? 2. On average, how many coaching sessions do you conduct per month?  - Of those, how many were scheduled, on demand, ad hoc? - And of those, what proportion are on Technical/HIA and what proportion are on management skills or other areas?  1. Which area of coaching do you find of most value (technical, management, other)? And, why? 2. How have you built the capacity of local geography staff? 3. How has your coaching changed overtime? 4. What has worked best for your coaching sessions: classroom or on-the-job supervision and coaching? | **Current Coaching Experience**   1. What areas do you coach on?  - Technical / (HIA) - Management - Others  1. Who do you coach? 2. On average, how many coaching sessions do you conduct per month?  - Of those, how many were scheduled, on demand, ad hoc? - And of those, what proportion are on Technical/HIA and what proportion are on management skills or other areas?  1. Which area of coaching do you find of most value (technical, management, other)? And, why? 2. How have you built the capacity of the City Health Coach/Service Provider? 3. How has your coaching changed overtime? 4. What has worked better for your coaching sessions, classroom or on-the-job supervision and coaching? 5. What benefit have you seen by being coached by TCI staff? 6. How confident are you to trickle down the coaching sessions? 7. From where do you receive coaching requests? Do you receive interdepartmental requests on coaching? | **Current Coaching Experience**   1. Do you coach? If yes, what areas do you coach on?  - Technical / (HIA) - Management - Others   If no, skip question6-10   1. How confident are you coaching (on a scale of 1-10)? What would make you feel more confident in your coaching ability? 2. What do you think is needed to build a good relationship between a coach and coachee? What reactions/responses do you receive from those you coach? 3. On average, how many coaching sessions do you conduct per month?  - Of those, how many were scheduled, on demand, ad hoc? - And of those, how many are on Technical/HIA, how many on management skills or other?  1. Which area of coaching do you find of most value (technical, management, other)? 2. How has your coaching changed overtime? 3. What has worked better for your coaching sessions, classroom or on-the-job supervision and coaching? 4. What benefit have you seen by being coached by TCI staff? |
| **Assess Technical Coaching**   1. What is your experience coaching technical vs. non-technical staff? 2. In your opinion, did you see an increase in the TCI program managers and program implementers family planning knowledge, attitudes, and practices following your coaching session? 3. Was that knowledge/skill retained over any extended period of time? 4. Have you seen an increase in HIA conducted by LG following your coaching support?^[[1]](#footnote-2)^ 5. In your experience, how long did it take to move between different stages – Lead, Assist, Observe for coaching city staff? 6. Have you seen an increase in the number of local governments receiving “observational” coaching for the city’s primary best practice interventions following your coaching support? 7. How is coaching different from advocacy efforts you do with political leadership? 8. Have you seen adoption and adaptation of family planning/AYSRH HIA incorporated into local policies, workplans, guidelines or standards following your coaching? Please provide examples. 9. Does the use of a support supervisor sheet help with assessing the quality of HIA implementation? 10. When are job aids used/referred to vs the detailed approach guidance on TCI U? 11. How frequently do coaches refer to TCI-U and for what primary reasons? 12. With step-down coaching, how do we monitor its quality and impact? 13. How can coaching with a select group influence diffusion to the entire city and beyond? Please provide an example, if you have one. | **Assess Technical Coaching**   1. What is the effect of TCI coaching on TCI program implementers’ family planning/AYSRH knowledge, attitudes, and practices? 2. Does use of support supervisor sheet help with assessing quality of HIA implementation? 3. When are job aids used/referred to vs the detailed approach guidance on TCI U? 4. How frequently do you refer to TCI U and for what primary reasons? 5. With step-down coaching, how to you monitor its quality and impact? 6. How can coaching with select groups influence diffusion to the entire city and beyond? 7. Do you feel confident that you have acquired all skills needed to implement TCI HIAs?  - Which HIAs or other areas do you still find challenging?  1. Have you had an opportunity to coach staff from other health facilities on TCI’s HIA?  - If so, were they able to understand the approach? - Have you had contact with them since the coaching session? - Did you refer them to TCI U?  1. What is the difference between coaching provided to TCI-supported health facility (HF) vs non-TCI HF? 2. In your experience, how long did it take you to move between the different stages of Lead – Assist – Observe? | **Assess Technical Coaching**   1. What are the effects of TCI coaching? How has it changed your family planning knowledge, attitudes, and practices? Probe: Have you seen a reduction in provider bias? 2. Does use of support supervisor sheet help with assessing quality of HIA implementation? 3. When are job aids used/referred to vs detailed approach guidance on TCI U? 4. How frequently do you refer to TCI U and for what primary reasons? 5. With step-down coaching, how to you monitor its quality and impact? 6. How can coaching with select groups influence diffusion to the entire city and beyond? 7. Do you feel confident that you have acquired all skills needed to implement TCI HIA?    - Which HIAs or other areas do you still find challenging? 8. Have you had an opportunity to coach staff from other health facilities on TCI’s HIA? 9. If so, were they able to understand the approach? 10. Have you had contact with them since the coaching session? 11. Did you refer them to TCI U |
| **Coaching on Effective Management**   1. How do we ensure that health system technocrats can be self-starters and self-directing (ensuring that they come up with their own creative solutions)? 2. In your opinion, have LG/DHD demonstrated greater FP/AYSRH coordination following your coaching? If so, was this sustained? 3. Have you seen an increase in the LG/DHD conducting quarterly RAISE assessments following your coaching? If so, has this been sustained? 4. Does the LG include more private sector partners in quarterly program implementation team meetings following your coaching? 5. Have you seen an increase in LG using data for decision-making to support FP/AYSRH implementation, following your coaching? Has this been consistent? 6. What is different in the geographies now because of TCI, compared to when we started? Probe: What hasn’t changed? 7. How has TCI coaching built/strengthened the health system beyond just HII? 8. What content are we missing on TCI_U that would help you as a coach? 9. How is coaching integrated in the daily work life of TCI staff and geography staff? 10. In your opinion, what makes certain geographies quick to adopt and implement TCI? Why do you think others take more time? | **Coaching on Effective Management**   1. How do we ensure that health system technocrats can be self-starters and self-directing (ensuring that they come up with their own creative solutions)? 2. How is coaching integrated in the daily work life of TCI staff and geography staff? 3. How has TCI coaching built/strengthened the health system beyond just HIA? 4. What content are we missing on TCI_U that would help you as a coach? 5. Do you feel confident that you can now lead resource allocation? 6. How has TCI helped you in your interactions with political leadership? 7. Do you participate in the PIT (Project Implementation Team) meetings (or any other monthly program review meeting started under TCI)? 8. Do you talk about coaching at those sessions? 9. What is different in the geography now because of TCI, compared to when we started? *Probe: What hasn’t changed?* 10. What makes certain geographies quick to adopt and implement TCI? Why do you think others take more time? 11. Are there areas that need more attention/ focus then others, what are they? | **Coaching on Effective Management**   1. How is coaching integrated into your daily work life? 2. How has TCI coaching built/strengthened the health system beyond just HIA? 3. How has TCI helped you in your interactions with political leadership? 4. Do you participate in the PIT (Project Implementation Team) meetings (or any other monthly program review meeting started under TCI)?  - Do you talk about coaching at those sessions?  1. What is different in your geography (or facility) now because of TCI, compared to when we started? *Probe: What hasn’t changed?*  - Are there areas that need more attention/focus then others, what are they? |
| **Recommendations for Improving Coaching & Sustainability**   1. Are there areas that need more attention/ focus than others, what are they? 2. In your opinion, what can be done to improve TCI coaching so that geographies can confidently transition from Lead Assist Observe (Startup, implement/ surge, pre-graduation, and post- graduation)? 3. What is the effect of TCI coaching on 4 pillars of sustainability -leadership, ownership, family planning commitments, demand, service, access, integration, and quality? 4. What role does coaching play to ensure operations and the gains experienced under TCI will live beyond TCI? | **Recommendations for Improving Coaching & Sustainability**   1. What role does coaching play to ensure operations and the gains experienced under TCI will live beyond TCI? 2. How can we improve TCI coaching so that you can confidently transition from program startup, implement/ surge, pre-graduation, and post- graduation? 3. If we were to leave tomorrow, could you carry on without TCI or would you require more support? If more support is needed, in what specific areas would you require more support? | **Recommendations for Improving Coaching & Sustainability**   1. What role does coaching play to ensure operations and the gains experienced under TCI will live beyond TCI? 2. If we were to leave tomorrow, could you carry on without TCI or would you require more support? If more support is needed, in what specific areas would you require more support? |

1. Questions highlighted in grey are linked to Results Framework on Coaching [↑](#footnote-ref-2)
