# Supplementary material for: Technical and management coaching for government institutions: Lessons learned and health systems transformations across 8 countries in sub-Saharan Africa and India
Source: PLOS Glob Public Health. 2025 Jan 3;5(1):e0004058. doi: 10.1371/journal.pgph.0004058 (PMC11698439; doi:10.1371/journal.pgph.0004058)
Supplement: S2 File — (DOCX) [file pgph.0004058.s002.docx]

## EAST AFRICA: COACHING STUDY **FGD** INTERVIEW GUIDE

| **Interview demographics** | | | |
| --- | --- | --- | --- |
| **#** | **Hub** |  | |
|  | **Interviewer’s name(s)** |  | |
|  | **Assistant / Note taker** |  | |
|  | **Interview date** |  | |
|  | **Interviewee role (Tick ALL that apply)** | Hub staff  Geography staff  Health care provider  Pharmacists | |
|  | **Total number participating per Interviewee role** | **Role** | **Totals** |
|  |  | Hub staff |  |
|  |  | Geography staff |  |
|  |  | Health care provider |  |
|  |  | Pharmacists |  |

**Welcome**

**START RECORDING**

Good morning/afternoon/evening and welcome to our virtual session.

Thanks for taking the time to join us and share feedback on your experience of coaching in the TCI program. My name is xxxxxxxxx and assisting me is xxxxxxxx. TCI stands for The Challenge Initiative, which is a program that works with local governments and the private health sector system to improve access to quality FP and AYSRH services for women and girls in the urban slums. TCI East Africa program implements in Tanzania, Kenya and Uganda. The main goal of this study is to explore further the successes, performance, challenges and innovations of The Challenge Initiative (TCI) programs coaching strategy, by determining the present situation around coaching, the local government awareness of the coaching opportunities and the application of coaching in day to day activities in family planning programming. The TCI coaching strategy (dubbed Sisi-kwa-sisi) is an innovative peer-to-peer learning strategy that uses counterpart coaching and mentoring to impart knowledge and skills in the workplace to meet a given objective.

We would like to talk with you today to explore your experiences, lessons learnt, preferences and any gaps for purposes of improving the delivery and implementation of the coaching approaches through adaptation as well as for documentation of which coaching content, practices, processes and tools are working well, or not working well.

**Consent Process**

As a group, we are going to go over the informed consent form before we start our focus

group to be sure that you understand why we are having this focus group and to be sure

that you voluntarily want to participate.

**Intro on focus groups**

- Ask the group if anyone has participated in a focus group before. Explain that focus

groups are being used more and more to gain information for better health programs.

- Alert them that they are the experts and we aim to learn from them – positive and negative feedback on their experiences and perspective is highly encouraged.
- We are not trying to get everyone to agree or achieve consensus, rather, we’re gathering

information. It is okay if you have different opinions and ideas than the other persons in

the group. We encourage everyone to participate actively.

**Focus group logistics and ground rules**

- Focus group will last about 1.5hours (90 minutes)
- Keep all your mics on mute unless called out to speak to allow one person to speak at a time
- Raise your hand by clicking the raised hand icon shown and you shall be called out to speak
- You can also post your response in the chat section
- The session will be recorded to help us gather more information about your responses and written notes will also be taken by the investigators for purposes of double checking the audio recordings.

*Ask the group if there are any questions before we get started, and address those questions.*

**Focus Group Questions**

*Discussion begins, make sure to give people time to think before answering the questions and don’t move too quickly.*

**Q1: Coaching learning curve: What has been your experience been providing coaching and How has your confidence in providing coaching changed over time? Compare when you started to current.**

Discussion points:

- What adjustments have you made
- What gaps still exists
- What would improve your coaching ability
- How is coaching integrated in the daily work life of TCI staff and geography staff?
- In your opinion, what makes certain geographies quick to adopt and implement TCI? Why do you think others take more time?

**Service delivery providers only: Coaching background. Integrate these discussions as part of question 1.**

**Discussion points:**

- Have you been coached or have you coached before?
- What interactions stood out for you during your coaching interactions (either as coach or coachee)?
- During your coaching interactions what role did TCI-U play?
- For those who have coached:
  - Have you coached staff from other health facilities and how did you go about it.

**Q2: Coaching experience: How has your coaching theme evolved over time?**

Discussion points:

- Coaching theme areas before and now. What are you coaching more of now compared to when we started?

Probe: Technical, Management / Leadership, Transitions/evolutions that have happened

- Was that knowledge/skill retained over any extended period of time?
- Experience on coaching technical versus non-technical staff
- What is different in the geographies now because of TCI or coaching, compared to when we started? *Probe:* What hasn’t changed?
- How has TCI coaching built/strengthened the health system beyond just HII?
- Have you seen an increase in HIA conducted by LG following your coaching support?
- How can coaching with a select group influence diffusion to the entire city and beyond? Please provide an example, if you have one.

**Q3: Coaching methodology: How do you go about identifying capacity gaps and prioritizing coaching solutions?**

Discussion points:

- Which tools do you use eg RAISE assessments?

*Probe:* What other sources in use

- What delivery method works best for your coaching?

*Probe:* classroom, on the job supervision/mentorship

- How frequently do you refer to TCI-U and for what primary reasons?
- When are job aids used/referred to vs the detailed approach guidance on TCI U?
- How many hours do you dedicate to coaching on a weekly basis?

*Probe:* What proportion would you assign to technical/HII and to program management skills?

- With cascade coaching, how do we monitor its quality and impact?

**Q4: Coaching recommendations: In your opinion, what can be done to improve TCI coaching so that geographies can confidently transition from Lead Assist Observe (Start-up, implement/ surge, pre-graduation, and post- graduation)?**

Discussion points:

- Are there areas that need more attention/ focus than others, what are they?
- What should be strengthened or added on TCI-U that would help you as a coach?
- What do you think is needed to build a good relationship between a coach and coachee?

TCI STAFF ONLY

**Q5: Coaching relationship with Gates Institute team: Has it been useful and which aspects of your work has it influenced?**

Discussion points:

- What is the main focus of this coaching?

Probe: Technical or non-technical

- Do you feel you need more support from Global?

Probe: What type of support?

- What cross hub learnings, if any, have changed the way you coach or work in general?
- In what ways, if any, has Global helped in your professional development?
- Does Global add value to technical areas of your work? If it does, can you please provide an example?

**TCI EA FIELD STAFF: FOCUS GROUP DISCUSSION**

|  | FGD date |  |
| --- | --- | --- |
|  | FGD facilitator |  |
|  | FGD Note taker |  |

**PARTICIPANT’S BACKGROUND**

| Background | | |
| --- | --- | --- |
| # | Hub | East Africa |
|  | Interviewee supporting area (Tick only one) | Hub ____________________ participants  Country_________________________ participants  Geography_________________________ participants |
|  | Interviewee Role | Hub leadership _____________________________ participants  Hub Technical management (FP, AY, M&E, Learning) ______ participants  Country Leadership/Country Lead, _____________ participants  Country technical management (FP, AY, M&E, Learning) _________ participants  Geography manager ________________ participants |

**CONSENT**

**GUIDE QUESTIONS**

| Q . NU | Question | Response guidance | Skip guidance |
| --- | --- | --- | --- |
| **Coaching history and support** | | | |
|  | Have you attended any session/ training on coaching methodologies in the past? | Yes  No | If No Skip to 1b |
|  | 1. If yes, who conducted the training? |  |  |
|  | 1. If no, do you feel a need to attend such a course? |  |  |
|  | 1. How confident are you coaching (on a scale of 1-10)? |  |  |
|  | 1. What would make you feel more confident in your coaching ability? |  |  |
|  | 1. Do you receive guidance from Hub on how to coach? | Yes  No | Ask only to City managers |
|  | 1. If yes, how often? | - Weekly - Monthly - Quarterly |  |
|  | 1. What is your experience of coaching you receive from GI/ Global TCI team? |  | Ask to hub staff, For non-hub staff skip to Q.5 |
|  | 1. Which aspects of your work has this coaching improved? |  |  |
|  | 1. Is it coaching on HIA, management, data analysis or other technical areas, please explain? |  |  |
|  | 1. Do you feel you need more support from Global? If so, what type of support? |  |  |
|  | 1. What cross hub learnings, if any, have changed the way you coach or work in general? |  |  |
|  | 1. In what ways, if any, has Global helped in your professional development? |  |  |
|  | 1. Does Global add value to technical areas of your work? If it does, can you please provide an example? |  |  |
|  | What do you think is needed to build a good relationship between a coach and coachee? |  |  |
|  | What reactions/responses do you receive from those you coach? |  |  |
| **Current coaching experience** | | | |
|  | Since you were hired to today, what areas do you coach on? | 1. Technical/ (HIA) 2. Management 3. Others (provide description) |  |
|  | Who do you coach? |  |  |
|  | 1. On average, how many coaching sessions do you conduct per month? |  | If 0 skip to 10 |
|  | 1. Of those, how many were scheduled, on demand, ad hoc? |  |  |
|  | 1. And of those, what proportion are on Technical/HIA and what proportion are on management skills or other areas? |  |  |
|  | Which area of coaching do you find of most value? And, why? | 1. Technical/ (HIA) 2. Management 3. Others (provide description) |  |
|  | How have you built the capacity of local geography staff? |  |  |
|  | How has your coaching changed overtime? |  |  |
|  | What has worked best for your coaching sessions: | 1. Classroom 2. On-the-job supervision and coaching? |  |
| **Assess Technical Coaching** | | | |
|  | What is your experience coaching technical vs. non-technical staff? |  |  |
|  | In your opinion, did you see an increase in the TCI program managers and program implementers family planning knowledge, attitudes, and practices following your coaching session? |  |  |
|  | Was that knowledge/skill retained over any extended period of time? |  |  |
|  | Have you seen an increase in HIA conducted by LG following your coaching support? |  |  |
|  | In your experience, how long did it take to move between different stages – Lead, Assist, Observe for coaching city staff? |  |  |
|  | Have you seen an increase in the number of local governments receiving “observational” coaching for the city’s primary best practice interventions following your coaching support? |  |  |
|  | How is coaching different from advocacy efforts you do with political leadership? |  |  |
|  | Have you seen adoption and adaptation of family planning/AYSRH HIA incorporated into local policies, workplans, guidelines or standards following your coaching? Please provide examples. |  |  |
|  | Does the use of a support supervisor sheet help with assessing the quality of HIA implementation? |  |  |
|  | When are job aids used/referred to vs the detailed approach guidance on TCI U? |  |  |
|  | How frequently do coaches refer to TCI-U and for what primary reasons? |  |  |
|  | With step-down coaching, how do we monitor its quality and impact? |  |  |
|  | How can coaching with a select group influence diffusion to the entire city and beyond? Please provide an example, if you have one. |  |  |
| **Coaching on Effective Management** | | | |
|  | How do we ensure that health system technocrats can be self-starters and self-directing (ensuring that they come up with their own creative solutions)? |  |  |
|  | In your opinion, have LG/DHD demonstrated greater FP/AYSRH coordination following your coaching? If so, was this sustained? |  |  |
|  | Have you seen an increase in the LG/DHD conducting quarterly RAISE assessments following your coaching? If so, has this been sustained? |  |  |
|  | Does the LG include more private sector partners in quarterly program implementation team meetings following your coaching? |  |  |
|  | Have you seen an increase in LG using data for decision-making to support FP/AYSRH implementation, following your coaching? Has this been consistent? |  |  |
|  | What is different in the geographies now because of TCI, compared to when we started? Probe: What hasn’t changed? |  |  |
|  | How has TCI coaching built/strengthened the health system beyond just HII? |  |  |
|  | What content are we missing on TCI_U that would help you as a coach? |  |  |
|  | How is coaching integrated in the daily work life of TCI staff and geography staff?  In your opinion, what makes certain geographies quick to adopt and implement TCI? Why do you think others take more time? |  |  |
| **Recommendations for Improving Coaching & Sustainability** | | | |
|  | Are there areas that need more attention/ focus than others, what are they? |  |  |
|  | In your opinion, what can be done to improve TCI coaching so that geographies can confidently transition from Lead Assist Observe (Start-up, implement/ surge, pre-graduation, and post- graduation)? |  |  |
|  | What is the effect of TCI coaching on 4 pillars of sustainability -leadership, ownership, family planning commitments, demand, service, access, integration, and quality? |  |  |
|  | What role does coaching play to ensure operations and the gains experienced under TCI will live beyond TCI? |  |  |
| **END** | | | |

**TCI EA GOVERNMENT OFFICIALS/HMT MEMBERS: FGD**

|  | FGD date |  |
| --- | --- | --- |
|  | FGD facilitator |  |
|  | FGD Note taker |  |

**PARTICIPANT’S BACKGROUND**

| Background | | |
| --- | --- | --- |
| # | Country |  |
|  | Interviewee supporting geography (List geography and number of participants) |  |
|  | Interviewee Role | Political leadership _____________________________ participants  Geography FP technical leadership ______ participants  Geography AYSRH technical leadership ______ participants  Sub Geography FP technical leadership ______ participants  Sub Geography FP technical leadership ______ participants |

**CONSENT**

**GUIDE QUESTIONS**

| Q . NU | Question | Response guidance | Skip guidance |
| --- | --- | --- | --- |
| **Coaching history and support** | | | |
|  | Have you attended any session/ training on coaching methodologies in the past? | Yes  No | If No Skip to 1b |
|  | 1. If yes, who conducted the training? |  |  |
|  | 1. If no, do you feel a need to attend such a course? |  |  |
|  | 1. How confident are you coaching (on a scale of 1-10)? |  |  |
|  | 1. What would make you feel more confident in your coaching ability? |  |  |
|  | 1. Do you receive guidance from Hub on how to coach? | Yes  No | Ask only to City managers |
|  | 1. If yes, how often? | - Weekly - Monthly - Quarterly |  |
|  | 1. What do you think is needed to build a good relationship between a coach and coachee? |  |  |
|  | 1. What reactions/responses do you receive from those you coach? |  |  |

| **Current coaching experience** |
| --- |

|  | What areas do you coach on? | 1. Technical/ (HIA) 2. Management 3. Others (provide description) |  |
| --- | --- | --- | --- |
|  | Who do you coach? |  |  |
|  | 1. On average, how many coaching sessions do you conduct per month? |  | Ask to at least half of the participants |
|  | 1. Of those, how many were scheduled, on demand, ad hoc? |  |  |
|  | 1. And of those, what proportion are on Technical/HIA and what proportion are on management skills or other areas? |  |  |
|  | Which area of coaching do you find of most value? And, why? | 1. Technical/ (HIA) 2. Management 3. Others (provide description) | Continue to probe on why for response provided |
|  | How have you built the capacity of local geography staff? |  |  |
|  | How has your coaching changed overtime? |  |  |
|  | What has worked better for your coaching sessions: classroom or on-the-job supervision and coaching? | 1. Classroom 2. On-the-job supervision and coaching? |  |
|  | What benefit have you seen by being coached by TCI staff? |  |  |
|  | How confident are you to trickle down the coaching sessions? |  |  |
|  | From where do you receive coaching requests? Do you receive interdepartmental requests on coaching? |  |  |

| **Assess Technical Coaching** |
| --- |

|  | What is the effect of TCI coaching on TCI program implementers’ family planning/AYSRH knowledge, attitudes, and practices? |  |  |
| --- | --- | --- | --- |
|  | Does use of support supervisor sheet help with assessing quality of HIA implementation? |  |  |
|  | When are job aids used/referred to vs the detailed approach guidance on TCI U? |  |  |
|  | How frequently do you refer to TCI U and for what primary reasons? |  |  |
|  | With step-down coaching, how to you monitor its quality and impact? |  |  |
|  | How can coaching with select groups influence diffusion to the entire city and beyond? |  |  |
|  | Do you feel confident that you have acquired all skills needed to implement TCI HIAs? |  |  |
|  | Which HIAs or other areas do you still find challenging? |  |  |
|  | 1. Have you had an opportunity to coach staff from other health facilities on TCI’s HIA? |  |  |
|  | 1. If so, were they able to understand the approach? |  |  |
|  | 1. Have you had contact with them since the coaching session? |  |  |
|  | 1. Did you refer them to TCI U? |  |  |
|  | What is the difference between coaching provided to TCI-supported health facility (HF) vs non-TCI HF? |  |  |
|  | In your experience, how long did it take you to move between the different stages of Lead – Assist – Observe? |  |  |

| **Coaching on Effective Management** |
| --- |

|  | How do we ensure that health system technocrats can be self-starters and self-directing | (ensuring that they come up with their own creative solutions)? |  |
| --- | --- | --- | --- |
|  | How is coaching integrated in the daily work life of TCI staff and geography staff? |  |  |
|  | How has TCI coaching built/strengthened the health system beyond just HIA? |  |  |
|  | What content are we missing on TCI-U that would help you as a coach? |  |  |
|  | Do you feel confident that you can now lead resource allocation? |  |  |
|  | How has TCI helped you in your interactions with political leadership? |  |  |
|  | Do you participate in the PIT (Project Implementation Team) meetings (or any other monthly program review meeting started under TCI)? |  |  |
|  | Do you talk about coaching at those sessions? |  |  |
|  | What is different in the geography now because of TCI, compared to when we started? | *Probe: What hasn’t changed?* |  |
|  | What makes certain geographies quick to adopt and implement TCI? Why do you think others take more time? |  |  |
|  | Are there areas that need more attention/ focus then others, what are they? |  |  |

| **Recommendations for Improving Coaching & Sustainability** |
| --- |

|  | What role does coaching play to ensure operations and the gains experienced under TCI will live beyond TCI? |  |  |
| --- | --- | --- | --- |
|  | How can we improve TCI coaching so that you can confidently transition from program startup, implement/ surge, pre-graduation, and post- graduation? |  |  |
|  | If we were to leave tomorrow, could you carry on without TCI or would you require more support? |  |  |
|  | If more support is needed, in what specific areas would you require more support? |  |  |

| **END** |
| --- |

**TCI EA SERVICE PROVIDERS: FGD**

|  | FGD date |  |
| --- | --- | --- |
|  | FGD facilitator |  |
|  | FGD Note taker |  |

**PARTICIPANT’S BACKGROUND**

| Background | | |
| --- | --- | --- |
| # | Country |  |
|  | Interviewee supporting geography (List geography and number of participants by ownership | 1. Geography ______________# Public _________ # Private |
|  | Interviewee Role | Health Facility leadership/in charge__________________ participants  FP service provider ______ participants  AYSRH service provider ______ participants  Pharmacist/Pharm technician ______ participants |

**CONSENT**

**GUIDE QUESTIONS**

| Q . NU | Question | Response guidance | Skip guidance |
| --- | --- | --- | --- |
| **Coaching history and support** | | | |

|  | Have you been coached on any family planning interventions? Probe: | Yes  No | If No Skip to 1b |
| --- | --- | --- | --- |
|  | If yes, what interventions have you been coached on? | probe |  |
|  | Did you find the coaching useful?  Probe: what was useful? What wasn’t? |  |  |
|  | Are you aware of TCI University? |  |  |
|  | Did your coach refer you to TCI University? |  |  |
|  | 1. Have you ever requested coaching? And, to whom have you requested it from? Probe: |  |  |
|  | 1. How long did it take for the coach to respond to your request? |  |  |
|  | What has changed in your routine tasks since you received coaching? |  |  |
| **Current coaching experience** | | | |
|  | Do you coach? | Yes  No | If no skip to 13 |
|  | If yes, what areas do you coach on? | 1. Technical/ (HIA) 2. Management 3. Others (provide description) |  |
|  | How confident are you coaching (on a scale of 1-10)? What would make you feel more confident in your coaching ability? |  |  |
|  | 1. What do you think is needed to build a good relationship between a coach and coachee? |  |  |
|  | 1. What reactions/responses do you receive from those you coach? |  |  |
|  | 1. On average, how many coaching sessions do you conduct per month? |  | Ask to at least half of the participants |
|  | 1. Of those, how many were scheduled, on demand, ad hoc? |  |  |
|  | 1. And of those, how many are on Technical/HIA and how many are on management skills or other areas? |  |  |
|  | Which area of coaching do you find of most value? And, why? | 1. Technical/ (HIA) 2. Management 3. Others (provide description) | Continue to probe on why for response provided |
|  | How has your coaching changed overtime? |  |  |
|  | What has worked better for your coaching sessions, classroom or on-the-job supervision and coaching? |  |  |
|  | What benefit have you seen by being coached by TCI staff? |  |  |

| **Assess Technical Coaching** |
| --- |

|  | 1. What are the effects of TCI coaching? |  |  |
| --- | --- | --- | --- |
|  | 1. How has it changed your family planning knowledge, attitudes, and practices? Probe: |  |  |
|  | 1. Have you seen a reduction in provider bias? |  |  |
|  | Does use of support supervisor sheet help with assessing quality of HIA implementation? |  |  |
|  | When are job aids used/referred to vs detailed approach guidance on TCI U? |  |  |
|  | How frequently do you refer to TCI U and for what primary reasons? |  |  |
|  | With step-down coaching, how to you monitor its quality and impact? |  |  |
|  | How can coaching with select groups influence diffusion to the entire city and beyond? |  |  |
|  | 1. Do you feel confident that you have acquired all skills needed to implement TCI HIA? |  |  |
|  | 1. Which HIAs or other areas do you still find challenging? |  |  |
|  | 1. Have you had an opportunity to coach staff from other health facilities on TCI’s HIA? |  |  |
|  | 1. If so, were they able to understand the approach? |  |  |
|  | 1. Have you had contact with them since the coaching session? |  |  |
|  | 1. Did you refer them to TCI U |  |  |

| **Coaching on Effective Management** |
| --- |

|  | How is coaching integrated into your daily work life? |  |  |
| --- | --- | --- | --- |
|  | How has TCI coaching built/strengthened the health system beyond just HIA? |  |  |
|  | 1. Do you participate in the PIT (Project Implementation Team) meetings (or any other monthly program review meeting started under TCI)? |  |  |
|  | 1. Do you talk about coaching at those sessions? |  |  |
|  | How has TCI helped you in your interactions with political leadership? |  |  |
|  | 1. What is different in your geography (or facility) now because of TCI, compared to when we started? *Probe: What hasn’t changed?* |  |  |
|  | 1. Are there areas that need more attention/focus then others, what are they? |  |  |

| **Recommendations for Improving Coaching & Sustainability** |
| --- |

|  | What role does coaching play to ensure operations and the gains experienced under TCI will live beyond TCI? |  |  |
| --- | --- | --- | --- |
|  | If we were to leave tomorrow, could you carry on without TCI or would you require more support? If more support is needed, in what specific areas would you require more support? |  |  |

| **END** |
| --- |
