# Supplementary material for: Technical and management coaching for government institutions: Lessons learned and health systems transformations across 8 countries in sub-Saharan Africa and India
Source: PLOS Glob Public Health. 2025 Jan 3;5(1):e0004058. doi: 10.1371/journal.pgph.0004058 (PMC11698439; doi:10.1371/journal.pgph.0004058)
Supplement: S3 File — (DOCX) [file pgph.0004058.s003.docx]

| **GUIDE D’INTERVIEW DES FGD: EXÉCUTANTS DU SYSTÈME DE SANTÉ (** | |
| --- | --- |
| Nom, poste et département de la **personnes interviewées** : |  |
| Ville (municipalité/système de santé) : |  |
| Noms de l’enquêteur (& personne chargée de la prise de notes) : |  |
| Date et heure de l’entretien : |  |
| Modalité de l’entretien : | Appel téléphonique/internet __ /autre _________ (*entourez et/ou remplissez une option*) |

1. ***Introduction et but de l’étude***
   Merci d’avoir accepté de participer à cette étude. Nous souhaitons comprendre comment les municipalités et les systèmes de santé en Afrique de l’Ouest Francophone (AOF) peuvent rendre la planification familiale et les services de santé sexuelle et reproductive des adolescents et des jeunes plus disponibles et plus accessibles. Nous interviewons différentes parties prenantes, particulièrement les dirigeants et les gérants qui travaillent dans les municipalités (communes/mairies) ou avec les systèmes de santé, ou encore dans les établissements de santé, comme c’est votre cas. Nous aimerions parler du travail de votre municipalité dans le domaine de la santé et la planification familiale, et de votre expérience de TCI. Notre but est de découvrir les meilleures manières dont les municipalités et les systèmes de santé en AOF peuvent renforcer et mettre a echelle les programmes de planification familiale, et partager ces résultats plus largement.
2. ***Logistique de l’entretien***

Nous aimerions commencer par nous présenter et vous donner quelques informations sur cet entretien.

*a) Présentation de l’équipe*

- The Challenge Initiative (TCI) est un programme financé par la Fondation Bill & Melinda Gates pour soutenir les gouvernements locaux afin de renforcer et de mettre a echelle les programmes de planification familiale pour les femmes, les jeunes et les adolescents.
- L’équipe chargée des interviews est composée des membres de TCI à l’Université de Johns Hopkins et de chacune des quatre régions de TCI, y compris ici en AOF.

*b) Information sur l’entretien et votre consentement à y participer*

- *Durée :* L’entretien devrait durer environ 2h.
- *Anonymat :* Nous ne relierons aucune de vos informations à votre nom sans votre permission.
- *Clarification et consentement :* N’hésitez pas à me poser des questions si vous ne comprenez pas. Vous pouvez mettre fin à l’entretien quand vous voulez.
- *Notes et consentement à être enregistré(e) :* Nous prendrons des notes pendant l’entretien, et nous aimerions enregistrer notre conversation. Cela m’aidera à relire mes notes et les compléter pour qu’elles soient plus exactes. Puis-je enregistrer notre conversation? *[****Attendre le consentement oral.]***

1. **Questions du guide d’interview**

Merci d’avoir pris le temps de me recevoir aujourd’hui. Sachez qu’il n’y a pas de bonne ou de mauvaise réponse; c’est vous l’expert(e). N’hésitez pas à être aussi franc(he) que possible. Avant de commencer, avez-vous des questions? *[Questions et réponses, si nécessaire]*

*Général (Posez toujours cette question en premier. Elle vise à établir un lien de confiance.)*

J’aimerais commencer par vous poser quelques questions sur vous.

1. Pouvez-vous me parler un peu de vous? En particulier, depuis combien de temps travaillez-vous dans la santé et la planification familiale, et depuis combien de temps êtes-vous à votre poste actuel?

*Questions générales*

1. Dans les deux dernières années, quelles sont, selon vous, les raisons clés pour lesquelles votre système de santé (et votre municipalité) a été capable d’augmenter la disponibilité et l’accès aux programmes de planification familiale (pour les femmes, les jeunes et les adolescents) pour celles et ceux qui le désirent?

*Relances:*

- Voyez-vous d’autres raisons à cela?
- À votre avis, quelle est la manière la plus importante dont TCI a aidé votre système de santé à renforcer votre programme de planification familiale pour les femmes, les jeunes et les adolescents?

1. Dans les deux dernières années, votre système de santé a-t-il fait des efforts particuliers pour renforcer votre programme de planification familiale pour les adolescents et les jeunes? Si oui, quels sont-ils? (Si oui, relancez pour mieux comprendre l’implication de TCI).

**INTERVENTIONS ET INTERVENANTS (TCI)**

*Adoption des interventions à haut impact en matière de planification familiale pour les femmes, jeunes, et adolescents) par la le système de santé*

1. Dans les deux dernières années, quelles interventions à haut impact spécifiques dans le domaine de la planification familiale pour les femmes, les adolescents et les jeunes le système de santé a-t-il adopté? (introduites par TCI)? Lesquelles ont été inscrites dans le plan de travail de votre système de santé et institutionnalisées dans le programme de planification familiale? (*Ne relancez que si nécessaire:* des interventions liées à la prestation de services? à la création de la demande? au plaidoyer? à la recherche, suivi et évaluation?) Comment cela s’est-il passé?

*Relance:*

- Quels sont, selon vous, les résultats de l’adoption de ces interventions à haut impact par le système de santé?

1. Une fois que votre système de santé a décidé d’adopter une intervention à haut impact en particulier (comme *l’ISBC*, les Journées Spéciales PF, les orientations du site complet, etc.), comment se coordonne-t-il avec d’autres acteurs (la municipalité, etc.) pour la mettre en œuvre et l’incorporer à son travail régulier? Utilisez un exemple d’intervention que votre système de santé a adoptée. (Si *nécessaire*: En d’autres termes, quelles sont les étapes clés que le système de santé devraient suivre pour maximiser la réussite de l’adoption et de la mise en œuvre de l’intervention dans le système de santé de manière continue?)

*Ne relancez que si nécessaire:*

- Quelles sont les difficultés communes rencontrées par le système de santé en travaillant avec la municipalité pour mettre en œuvre les interventions à haut impact?
- Comment le personnel du système de santé essaie-t-il de surmonter ces difficultés?

1. Le système de santé a-t-il modifié l'une des interventions à haut impact introduites par TCI ou d'autres pour qu’elle corresponde mieux au contexte local du système de santé? (Par exemple, lorsque vous introduisez pour la première fois une intervention à haut impact au niveau du district ou de l'établissement de santé, ou lorsque la municipalité développe son budget et son plan de travail ou implique des acteurs communautaires?). Si oui, donnez un exemple.

***Adoption de nouveaux approches de collaboration avec la municipalite***

1. Dans les deux dernières années, TCI vous a-t-elle aidé(e) à changer vos processus ou vos systèmes pour renforcer votre programme de planification familiale? Pour renforcer la collaboration avec la municipalité? *Relancez si nécessaire :* Par exemple, avez-vous vu des changements dans les approches manageriales ou processus du système de santé, comme par exemple l’utilisation des données pour la gestion du programme, l’examen de la qualité des données, coordination avec la municipalite (et son plan de travail/budgétisation, le financement de la planification familiale - lignes budgétaires), la coordination et la gestion des partenaires, l’adoption des lignes directrices nationales, etc.?

*Relance :* Si oui, pouvez-vous décrire comment ces changements ont eu lieu en reprenant l’un des exemples que vous venez de citer?

1. Le fait de travailler avec TCI a-t-il changé votre propre manière de faire votre travail? Si oui, comment?

***Institutionalisation des interventions a hauts impacts et des approches managériales***

Les questions suivantes portent sur la probabilité de durabilité et d’institutionnalisation des interventions à haut impact et des processus que le système de santé a adoptés.

1. Nous savons que les programmes des partenaires externes ne durent pas pour toujours. Si TCI se termine demain, quelles interventions de planification familiale à haut impact introduites par TCI et quelles approches manageriales, ont, selon vous, le **plus de chances de continuer** dans le système de santé sur le long terme? Lesquelles ont **le plus de chances de disparaître? Pourquoi?** (*Ne relancez que si nécessaire :* En d’autres termes, depuis votre point de vue, dans quelle mesure les interventions à haut impact sont-elles intégrées de manière durable dans le travail quotidien du système de santé?)

***Caractéristiques de l’intervenant (TCI)***

Maintenant, j’ai des questions pour savoir si la manière dont TCI travaille vous a aidé(e) à introduire et adopter de nouvelles approches.

1. En quoi l’approche de TCI - sa façon de travailler avec vous et le système de santé pour élargir le programme de planification familiale - a affecté l’introduction des interventions à haut impact? (Cela pourrait être soit la manière dont ils travaillent avec leurs collègues dans le système de santé ou à la municipalité, soit leurs caractéristiques individuelles).

*Relances si nécessaire:*

- Qu’est-ce qui pourrait être renforcé? En quoi est l’approche de TCI différente ou la même que de celle d’autres partenaires?

***PROCESSUS : Systèmes gouvernementaux et processus de coordination***

A présent, je vais vous poser des questions sur les aspects spécifiques qui caractérisent la manière dont le système de santé renforce ses systèmes et coordonne avec la municipalité (ses processus, ses mécanismes de coordination) pour accroitre la disponibilité du programme de planification familiale pour les les femmes, les adolescents et les jeunes qui le désirent.

***Utilisation des données pour la gestion des programmes***

1. Pouvez-vous décrire de manière générale comment le système de santé utilise les données pour administrer et améliorer son programme de planification familiale? Quels changements, le cas échéant, avez-vous vus dans la manière dont le système de santé a utilisé les données ces deux dernières années? (*Relancez si nécessaire:*  En d’autres termes, décrivez comment votre système de santé assure le suivi et évalue la qualité et l’efficacité de son programme de planification familiale, utilise les données pour identifier et répondre aux problèmes et sélectionner des stratégies, etc.).

*Relances:*

- Comment le système de santé suit-il les progrès dans la prestation des services aux jeunes et aux adolescents? Comment, le cas échéant, les données désagrégées par âge ont-elles été utilisées pour améliorer les programmes?
- À votre avis, comment TCI a-t-elle contribué à ces changements?

***Coordination***

1. Quels processus de planification et coordination du système de santé sont les plus déterminants pour gérer les programmes de planification familiale du système de santé, y compris tous les partenaires PF? Pourquoi? (*Relancez si nécessaire:*  Par exemple, les processus de planification et coordination du système de santé en matière de politique, prestation de services, création de la demande (sensibilisation, communication, IECC), utilisation des données pour la gestion, les comités, les groupes inter-départements, les groupes de travail pour l’amélioration des données, etc.)?

*Relance:* Comment ces processus ont-ils changé ces deux dernières années, le cas échéant?

*Relancez si ce n’est pas mentionné :* De quelles manières, le cas échéant, TCI a-t-elle contribué à ces processus de coordination?

1. Quel est le niveau de coordination (de la mise en oeuvre du programme PF) avec la municipalité? Comment la coordination est-elle mené?

**FACTEURS CONTEXTUELS INTERNES (***Priorité de la planification familiale pour le système de santé, alignement du partenariat TCI avec les politiques et priorités du système de santé au niveau national et local, etc.)*

Maintenant, je vais vous poser quelques questions sur le niveau de priorité de la planification familiale pour votre système de santé.

1. Pouvez-vous parler du niveau de priorité que votre système de santé accorde à la planification familiale? Comment le niveau de priorité de la planification familiale se compare-t-il à celui accordé aux autres domaines de la santé? (En quoi cela a-t-il changé ces deux dernières années? Qu’est-ce qui a contribué à ce changement?)

*Relancez si ce n’est pas mentionné :* Dans quelle mesure, le cas échéant, TCI a-t-elle joué un rôle dans ce changement?

*Leadership/soutien (voir aussi les priorités du gouvernement)*

1. Dans votre système de santé durant ces deux dernières années, quelle a été l’importance du rôle joué par les champions et supporteurs de la PF pour élargir la disponibilité et l’accès de la planification familiale et des programmes pour les femmes, les adolescents et les jeunes? Quel a été ce rôle des champions? (Ca peut être leaders dans le gouvernement, leaders religieux, leaders communautaires, etc.) À quels moments spécifiques le soutien de ces champions est-il le plus utile?

- Relancez si nécessaire : Et les **champions internes** à votre système de santé et votre municipalité? (comme le soutien des responsables politiques et des technocrates : les Conseillers, le Secrétaire Exécutif, le Coordinateur de la ville, etc.)
- Relancez si nécessaire : Et les **champions externes**? (champions en dehors du gouvernement comme les chefs religieux, les membres du groupe de plaidoyer, les membres d’un groupe de travail, etc.)

***Effets inattendus du partenariat entre la municipalité ou le système de santé et TCI***

Ces questions cherchent à savoir si vous avez vu un effet inattendu du partenariat entre votre municipalité ou système de santé avec TCI.

1. Quels effets additionnels ou inattendus avez-vous vu découler du partenariat entre votre système de santé (et la municipalité) et TCI? – au-delà d’élargir l’accès à la planification familiale et la planification pour les femmes et les jeunes intéressés ? (ex. Y a t-il d’autres domaines – de santé, de techniques, de managériales, ou de coordination - qui ont été affecté?)

*Relance si n*é*cessaire:*  Comme sur les systèmes de santé, sur la gestion d’autres programmes de santé, ou sur l’atteinte d’un objectif plus large dans l’agenda du système de santé?

(*Relancez si l’informateur ne comprend pas:* Par exemple, des effets inattendus pourraient comprendre des changements de directives/politiques, ou encore l’utilisation améliorée des données pour la prise de décision, une meilleure qualité des services ou un meilleur état de préparation des établissements de santé; la diffusion des interventions introduites par TCI dans d’autres sites (non TCI); une meilleure coordination des parties prenantes et des exécutants du gouvernement; moins de ruptures de stocks; l’intégration des services; etc.)

**POUR CONCLURE:**

1. À l’avenir, qu’est-ce que le système de santé devra faire de plus pour renforcer et ameliorer la mise en œuvre du programme de planification familiale pour le rendre durable?
2. En regardant en arrière, avec ce vous savez maintenant: Si vous deviez recommencer votre partenariat avec TCI depuis le début, qu’est-ce que vous feriez différemment?
3. Avez-vous d’autres commentaires dont vous voudriez nous faire part? (Sommes-nous passé à côté de points importants qui expliquent comment le système de santé et la municipalité peuvent élargir durablement l’accès à la planification familiale sur le long terme?)

Merci beaucoup d’avoir participé à cet entretien. Nous vous sommes très reconnaissants de nous avoir donné de votre expertise, de vos perspectives et de votre temps. Nous espérons que nous pourrons vous recontacter si nous avons besoin de clarifier certaines questions.

Guide d’entretien de l’étude sur l’approche d’engagement des villes de The Challenge Initative : une étude de cas sur le coaching

**Prénom et Nom de l’interviewé:**

**Titre du poste occupé :**

**Contacts :**

**Ville d’intervention :**

**Pays :**

| Prestataires de santé mettant en œuvre les approches TCI |
| --- |
| **Antécédent de coaching et soutien**   1. Avez-vous déjà été coaché(e) sur des interventions de planification familiale ? Relance : Si oui, sur quelles interventions avez-vous été coaché(e) ? 2. Avez-vous trouvé le coaching utile ? Relance : qu’est-ce qui a été utile ? Qu’est-ce qui ne l’a pas été ? 3. Connaissez-vous TCI University ? Votre coach vous a-t-il/elle orienté (e) vers TCI University ? 4. Avez-vous déjà fait une demande de coaching? À qui ? Relance : En combien de temps le/la coach a-t-il/elle répondu à votre demande ? 5. Qu’est-ce qui a changé dans vos tâches régulières depuis que vous avez été coaché(e) ? |
| **Expérience actuelle du coaching**   1. Coachez-vous ? Si oui, dans quels domaines ?  - Technique / Approches de haut impact - Management - Autres   Si non, sautez les questions 6-10   1. À quel point vous sentez-vous à l’aise en coachant (sur une échelle de 1 à 10) Qu’est-ce qui permettrait de vous sentir plus à l’aise concernant votre capacité à coacher ? 2. Que faut-il, selon vous, pour construire une bonne relation entre un coach et ses coachés ? Quelles réactions/réponses recevez-vous des personnes que vous coachez ? 3. En moyenne, combien de sessions de coaching conduisez-vous par mois ?  - Sur ces sessions mensuelles, combien sont programmées, sur demande, et ad hoc ? - Et sur ces mêmes sessions mensuelles, quelle est la proportion des sessions sur des sujets techniques/ approches de haut impact, sur des compétences de management/ gestion, et sur d’autres domaines ?  1. Quel domaine du coaching est de plus grande valeur (technique, management ou autre) selon vous ? Pourquoi ? 2. Votre coaching a-t-il changé avec le temps ? 3. Qu’est-ce qui a le mieux fonctionné pour vos sessions de coaching : les cours en salle de classe ou la supervision et coaching au travail ? 4. Quel bénéfice avez-vous observé après avoir été coaché(e) par le personnel de TCI ? |
| **Evaluation du coaching technique**   1. Quels sont les effets du coaching de TCI ? En quoi a-t-il changé vos connaissances, attitudes et pratiques de planification familaile/ SSRJA ? Relance : Avez-vous observé une baisse des préjugés chez les prestataires de santé ? 2. L’utilisation d’une fiche de supervision accompagnante (*support supervisor sheet*) est-elle utile pour évaluer la qualité de la mise en œuvre des approches de haut impact ? 3. Quand sont utilisées/ référées les aides mémoire vs. une approche de conseil plus détaillée sur TCI U ? 4. À quelle fréquence orientez-vous vos coachés vers TCI U et pour quelles raisons principales ? 5. Comment suivre la qualité et l’impact du coaching quand il est « en retrait » ? 6. Comment le coaching d’un groupe sélectionné peut-il influencer la diffusion d’approches dans toute la ville et au-delà ? Veuillez donner un exemple, si vous en avez. 7. Avec quelle certitude pensez-vous avoir acquis toutes les compétences nécessaires pour mettre en œuvre les approches à haut impact de TCI ? 8. Quelles approches ou domaines représentent encore un défi pour vous ? 9. Avez-vous eu l’opportunité de coacher du personnel d’autres structures de santé sur les approches à haut impact de TCI ? 10. Si oui, ont-ils compris l’approche ? 11. Avez-vous été en contact avec eux depuis votre session de coaching ? 12. Les avez-vous orientés vers TCI U ? |
| **Coaching sur le management effiace**   1. De quelle manière le coaching est-il intégré à la vie quotidienne des agents de TCI et des géographies ? 2. En quoi le coaching de TCI a-t-il renforcé le système de santé au-delà des interventions de haut impact ? 3. De quelle manière TCI vous a-t-elle aidé(e) dans vos interactions avec les dirigeants politiques ? 4. Participez-vous aux réunions de l’équipe de mise en œuvre du projet (ou toute autre réunion mensuelle initiée sous le programme TCI) ?  - Parlez-vous du coaching à ces réunions ?  1. Qu’est-ce qui est aujourd’hui différent dans votre géographie (ou structure de santé) grâce à TCI, en comparaison au moment où nous avons commencé ? *Relance : qu’est-ce qui n’a pas changé ?*    - Certains domaines requèrent-ils plus d’attention que d’autres ? Lesquels ? |
| **Recommendations pour améliorer le coaching et la durabilité des programes**   1. Quel rôle le coaching doit-il remplir pour s’assurer que les opérations et les gains obtenus sous TCI perdurent au-delà du programme ? 2. Si nous devions partir demain, pourriez-vous continuer sans TCI ou auriez-vous besoin dep lus de soutien ? Si plus de soutien est nécessaire, dans quels domaines spécifiques ? |

Guide d’entretien de l’étude sur l’approche d’engagement des villes de The Challenge Initative : une étude de cas sur le coaching

**Prénom et Nom de l’interviewé :**

**Titre du poste occupé :**

**Contacts :**

**Ville d’intervention :**

**Pays :**

| **Agents de terrain des plateformes d’accélération TCI** |
| --- |
| **Antécédent de coaching et soutien**   1. Avez-vous déjà assisté à une session/ formation sur les méthodologies de coaching par le passé ?  - Si oui, qui a conduit la formation ? - Si non, sentez-vous le besoin d’une telle formation ?  1. À quel point vous sentez-vous à l’aise en coachant (sur une échelle de 1 à 10)? Qu’est-ce qui pourrait vous rendre plus à l’aise concernant votre capacité à coacher ? 2. [Pour les gestionnaires des villes] Recevous-vous des conseils de la Plateforme d’accélération sur comment coacher ? Si oui, à quelle fréquence ?  - Une fois par semaine - Une fois par mois - Une frois par trimestre  1. [Pour les Plateformes] Quelle est votre expérience du coaching que vous avez reçu de l’IG/équipe de TCI Global ?  - Quels aspects de votre travail ce coaching a-t-il améliorés ? - Est-ce du coaching sur les approches de haut impact, la gestion, l’analyse de données ou un autre domaine technique ? Veuillez expliquer. - Pensez-vous nécessaire plus de soutien de TCI Global ? Si oui, quel type de soutien ? - Quels apprentissages inter-plateformes, s’il y en a, ont changé la manière dont vous coachez ou travaillez en général ? - De quelles manières, le cas échéant, TCI Global a-t-il aidé votre développement professionnel ? - TCI Global apporte-t-il une valeur ajoutée aux domaines techniques de votre travail ? Si c’est le cas, pourriez-vous donner un exemple ? - Que faut-il, selon vous, pour construire une bonne relation entre un coach et ses coachés ? Quelles réactions/réponses recevez-vous des personnes que vous coachez ? |
| **Expérience actuelle du coaching**   1. Depuis que vous avez été embauché(e) jusqu’à aujourd’hui, dans quels domaines coachez-vous ?  - Technique / Approches de haut impact - Management - Autres  1. Qui coachez-vous ? 2. En moyenne, combien de sessions de coaching conduisez-vous par mois ?  - Sur ces sessions mensuelles, combien sont programmées, sur demande, et ad hoc ? - Et sur ces mêmes sessions mensuelles, quelle est la proportion des sessions sur des sujets techniques/ approches de haut impact, sur des compétences de management/ gestion, et sur d’autres domaines ?  1. Quel domaine du coaching est de plus grande valeur (technique, management ou autre) selon vous ? Pourquoi ? 2. De quelle manière avez-vous renforcé les capacités du personnel des géographies locales ? 3. De quelle manière votre coaching a-t-il changé avec le temps ? 4. Qu’est-ce qui a le mieux fonctionné pour vos sessions de coaching : les cours en salle de classe ou la supervision et coaching au travail ? |
| **Evaluation du coaching technique**   1. Quelle est votre expérience du coaching pour des profiles techniques vs. non techniques ? 2. Selon vous, les connaissances, attitudes et pratiques de planification familiale des gestionnaires et exécutants de programmes ont-elles augmenté suite à vos sessions de coaching ? 3. Ces connaissances/ compétences ont-elles été retenues sur une longue durée ? 4. Avez-vous observé une augmentation des approches à haut impact conduites par les gouvernements locaux suite à votre coaching?^[[1]](#footnote-1)^ 5. Selon votre expérience, combien de temps cela a-t-il pris de passer d’une phase de coaching à l’autre (« Lead », « Assist », « Observe ») pour les agents des villes ? 6. Avez-vous observé une augmentation du nombre de gouvernements locaux recevant du coaching « observationnel » pour les principales interventions de la ville suite à votre coaching ? 7. En quoi le coaching est-il différent des efforts de plaidoyer que vous conduisez auprès des dirigeants politiques ? 8. Avez-vous observé une adoption et adaptation des approches à haut impact de planification familiale/SSRJA incorporées aux politiques, plans de travail, directives et standards locaux suite à votre coaching ? Veuillez donner des exemples. 9. L’utilisation d’une fiche de supervision accompagnante (*support supervisor sheet*) est-elle utile pour évaluer la qualité de la mise en œuvre des approches de haut impact ? 10. Quand sont utilisées/ référées les aides mémoire vs. une approche de conseil plus détaillée sur TCI U ? 11. À quelle fréquence les coaches orientent-ils leurs coachés vers TCI U et pour quelles raisons principales ? 12. Comment suivre la qualité et l’impact du coaching quand il est « en retrait » ? 13. Comment le coaching d’un groupe sélectionné peut-il influencer la diffusion d’approches dans toute la ville et au-delà ? Veuillez donner un exemple, si vous en avez. |
| **Coaching sur le management effiace**   1. Comment veiller à ce que les technocrates du système de santé prennent des initiatives et soient déterminés (trouvent eux-mêmes des solutions créatives) ? 2. À votre avis, les gouvernements locaux ont-ils démontré une meilleure coordination autour de la PF/SSRJA suite à votre coaching ? Si oui, cela a-t-il été maintenu ? 3. Avez-vous observé une augmentation du nombre de gouvernements locaux conduisant des évaluations trimestrielles RAISE suite à votre coaching ? Si oui, cela a-t-il continué ? 4. Le gouvernement local inclut-il plus de partenaires du secteur privé aux réunions trimestrielles de l’équipe de mise en œuvre du programme suite à votre coaching ? 5. Avez-vous observé une augmentation du nombre de gouvernements locaux utilisant des données pour la prise de décision sur la mise en œuvre des interventions de PF/SSRJA suite à votre coaching ? Cela a-t-il été constant ? 6. Qu’est-ce qui est aujourd’hui différent dans les géographies grâce à TCI, en comparaison au moment où nous avons commencé ? Relance : Qu’est-ce qui n’a pas changé ? 7. En quoi le coaching de TCI a-t-il renforcé le système de santé au-delà des interventions de haut impact ? 8. Quels contenus font défaut sur TCI U et qui vous seraient utiles en tant que coach ?      1. De quelle manière le coaching est-il intégré à la vie quotidienne des agents de TCI et des géographies ? 2. Selon vous, qu’est-ce qui fait que certaines géographies adoptent et mettent en œuvre TCI rapidement ? Pourquoi, selon vous, d’autres prennent plus de temps ? |
| **Recommendations pour améliorer le coaching et la durabilité des programmes**   1. Certains domaines requèrent-ils plus d’attention que d’autres ? Lesquels ? 2. À votre avis, de quelle manière peut-on améliorer le coaching de TCI pour que les géographies puissent passer aisémment de la phase « Lead » à « Assist » à « Observe » ? (Démarrage, Mise en œuvre/ « Surge », pré-graduation et post- graduation) ? 3. Quel est l’effet du coaching de TCI sur les 4 pilliers de la durabilité (leadership, appropriation, engagements de planification familiale, et demande, service, accès, intégration et qualité ?) 4. Quel rôle le coaching doit-il remplir pour s’assurer que les opérations et les gains obtenus sous TCI perdurent au-delà du programme ? |

Guide d’entretien de l’étude sur l’approche d’engagement des villes de The Challenge Initative : une étude de cas sur le coaching

**Prénom et Nom de l’interviewé :**

**Titre du poste occupé :**

**Contacts :**

**Ville d’intervention :**

**Pays :**

| Agents du gouvernement (états, comtés, villes) travaillant en partenariat avec TCI |
| --- |
| **Antécédent de coaching et soutien**   1. Avez-vous dèjà assisté à une formation sur les méthodologies de coaching par le passé ?  - Si oui, qui a conduit la formation ? - Si non, sentez-vous le besoin d’une telle formation ?  1. À quel point vous sentez-vous à l’aise en coachant (sur une échelle de 1 à 10)? Qu’est-ce qui pourrait vous rendre plus à l’aise concernant votre capacité à coacher ? 2. À quelle fréquence demandez-vous du coaching de TCI?  - Une fois par semaine - Une fois par mois - Une fois par trimestre  1. Que faut-il, selon vous, pour construire une bonne relation entre un coach et ses coachés ? Quelles réactions/réponses recevez-vous des personnes que vous coachez ? |
| **Expérience actuelle du coaching**   1. Dans quels domaines coachez-vous ?  - Technique / Approches de haut impact - Management - Autres  1. Qui coachez-vous ? 2. En moyenne, combien de sessions de coaching conduisez-vous par mois ?  - Sur ces sessions mensuelles, combien sont programmées, sur demande, et ad hoc ? - Et sur ces mêmes sessions mensuelles, quelle est la proportion des sessions sur des sujets techniques/ approches de haut impact, sur des compétences de management/ gestion, et sur d’autres domaines ?  1. Quel domaine du coaching est de plus grande valeur (technique, management ou autre) selon vous ? Pourquoi ? 2. Comment avez-vous renforcé les capacités des coaches santé de la ville/ prestataires de santé ? 3. De quelle manière votre coaching a-t-il changé avec le temps ? 4. Qu’est-ce qui a le mieux fonctionné pour vos sessions de coaching : les cours en salle de classe ou la supervision et coaching au travail ? 5. Quel bénéfice avez-vous observé après avoir été coaché(e) par les agents de TCI ? 6. À quel point vous sentez-vous capable de transmettre et coaché d’autres personnes/ cadres inférieurs ? 7. D’où recevez-vous des demandes de coaching ? Recevez-vous des demandes de coaching inter-services ? |
| **Evaluation du coaching technique**   1. Quel est l’effet du coaching de TCI sur les connaissances, attitudes et pratiques de planification familaile/ SSRJA des exécutants de programmes TCI ? 2. L’utilisation d’une fiche de supervision accompagnante (*support supervisor sheet*) est-elle utile pour évaluer la qualité de la mise en œuvre des approches de haut impact ? 3. Quand sont utilisées/ référées les aides mémoire vs. une approche de conseil plus détaillée sur TCI U ? 4. À quelle fréquence orientez-vous vos coachés vers TCI U et pour quelles raisons principales ? 5. Comment suivre la qualité et l’impact du coaching quand il est « en retrait » ? 6. Comment le coaching d’un groupe sélectionné peut-il influencer la diffusion d’approches dans toute la ville et au-delà ? Veuillez donner un exemple, si vous en avez. 7. Avec quelle certitude pensez-vous avoir acquis toutes les compétences nécessaires pour mettre en œuvre les approches à haut impact de TCI ?  - Quelles approches ou domaines représentent encore un défi pour vous ?  1. Avez-vous eu l’opportunité de coacher du personnel d’autres structures de santé sur les approches à haut impact de TCI ?  - Si oui, ont-ils compris l’approche ? - Avez-vous été en contact avec eux depuis votre session de coaching ? - Les avez-vous orientés vers TCI U ?  1. Quelle est la différence entre du coaching fourni aux structures de santé soutenues par TCI et à celles qui ne sont pas soutenues par TCI ? 2. Selon votre expérience, combien de temps cela vous a pris de passer d’une phase de coaching à l’autre (« Lead – Assist – Observe ») ? |
| **Coaching sur le management effiace**   1. Comment veiller à ce que les technocrates du système de santé prennent des initiatives et soient déterminés (trouvent eux-mêmes des solutions créatives) ? 2. De quelle manière le coaching est-il intégré à la vie quotidienne des agents de TCI et des géographies ? 3. En quoi le coaching de TCI a-t-il renforcé le système de santé au-delà des interventions de haut impact ? 4. Quels contenus font défaut sur TCI U et qui vous seraient utiles en tant que coach ? 5. Pensez-vous avec certitude que vous avez maintenant la capacité de diriger l’allocation des ressources ? 6. De quelle manière TCI vous a-t-elle aidé(e) dans vos interactions avec les dirigeants politiques ? 7. Participez-vous aux réunions de l’équipe de mise en œuvre du projet (ou toute autre réunion mensuelle initiée sous le programme TCI) ? 8. Parlez-vous du coaching à ces réunions ? 9. Qu’est-ce qui est aujourd’hui différent dans votre géographie grâce à TCI, en comparaison au moment où nous avons commencé ? *Relance : Qu’est-ce qui n’a pas changé ?* 10. Selon vous, qu’est-ce qui fait que certaines géographies adoptent et mettent en œuvre TCI rapidement ? Pourquoi, selon vous, d’autres prennent plus de temps ? 11. Certains domaines requèrent-ils plus d’attention que d’autres ? Lesquels ? |
| **Recommendations pour améliorer le coaching et la durabilité des programes**   1. Quel rôle le coaching doit-il remplir pour s’assurer que les opérations et les gains obtenus sous TCI perdurent au-delà du programme ? 2. De quelle manière peut-on améliorer le coaching de TCI pour que vous puissiez passer aisémment de la phase « Lead » à « Assist » à « Observe » ? (Démarrage, Mise en œuvre/ « Surge », pré-graduation et post- graduation) ? 3. Si nous devions partir demain, pourriez-vous continuer sans TCI ou auriez-vous besoin de plus de soutien ? Si plus de soutien est nécessaire, dans quels domaines spécifiques ? |

1. Les questions soulignées en gris sont liées au Cadre de résultats du coaching [↑](#footnote-ref-1)
