# Supplementary material for: Technical and management coaching for government institutions: Lessons learned and health systems transformations across 8 countries in sub-Saharan Africa and India
Source: PLOS Glob Public Health. 2025 Jan 3;5(1):e0004058. doi: 10.1371/journal.pgph.0004058 (PMC11698439; doi:10.1371/journal.pgph.0004058)
Supplement: S4 File — (DOCX) [file pgph.0004058.s004.docx]

| **KEY INFORMANT INTERVIEW GUIDE: Government Officials (To be administered with CMO, ACMO, UHC, MOIC and other city level coaches)** | |
| --- | --- |
| **Interviewee** name, job title: |  |
| City: |  |
| Interviewer & Note taker name(s) (as applicable): |  |
| Interview date and time: |  |
| Interview modality: | phone/internet call (Zoom) /other _____________ (*circle and/or fill in one*) |
| Links to recording & transcript: |  |

**Introduction and study purpose**

[Introduce yourself—name, title, location].

Say: I am a member of The Challenge Initiative or TCI team. Thank you for agreeing to participate in this study about coaching. TCI seeks to better understand how coaching has evolved over the past four years, including the successes, challenges, and innovations of TCIHC’s approach to coaching. We are interviewing people in five cities and three states from a variety of stakeholder groups, including TCI staff, city and government leaders and managers, facility staff, and community mobilizers. We want to hear from you because of your experience and knowledge of how TCI has worked with the cities and states. Our aim is to have findings lead to improvement in our approach to working with government through technical assistance and coaching.

**परिचय तथा अध्ययन का उद्देश्य**

[अपना परिचय दें —नाम, किस पद पर काम करते हैं, स्थान].

कहें: मैं द चैलेंज इनिशिएटिव/टी.सी.आई. टीम का/की सदस्य हूँ . कोचिंग सम्बन्धी इस अध्ययन में भाग लेने के लिए धन्यवाद. TCI इस बात को बेहतर ढंग से समझना चाहता है की पिछले चार वर्षों में कोचिंग प्रक्रिया *तथा इसके LAO (लीड, असिस्ट, ओब्सर्व) मॉडल जिसमें पहले चरण में किसी कौशल/गतिविधि को करके दिखाया जाता है(लीड), उसके बाद दूसरे चरण में कोची को करने का अवसर देकर आवश्यकतानुसार तकनीकी या प्रबंधन सहयोग दिया जाता है (असिस्ट) अंत में तीसरे चरण में कोची सम्पूर्ण उत्तरदायित्व लेकर उस गतिविधि /कौशल को करता है और कोच ओब्सर्व करता है,* किस प्रकार विकसित हुआ. जिसमें इस मॉडल की सफलता, चुनौतियाँ तथा TCIHC के कोचिंग करने के तरीकों में किये गए अभिनव प्रयास शामिल हैं. इसके लिए हम तीन प्रान्तों के पांच शहरों के विभिन्न स्टेकहोल्डर्स के साक्षात्कार कर रहे हैं जिनमें TCI का स्टाफ, शहरी स्वास्थ्य विभाग के अधिकारी तथा कार्यकर्ता, स्वास्थ्य केंद्र स्तर का स्टाफ तथा समुदाय स्तर पर काम करने वाले कार्यकर्ता शामिल हैं. हम आपसे इसलिए बातचीत करना चाहते हैं क्योंकि आपके पास इस बात की जानकारी तथा अनुभव है कि TCI ने शहरों तथा राज्य के साथ किस प्रकार काम किया है. हमारा उद्देश्य उन निष्कर्षों तक पहुँचना है जिनसे हम सरकार के साथ काम करने के अपने तकनीकी सहायता और कोचिंग के तरीके को और अच्छा बना सकें.

**Informed Consent and Confidentiality Statement (Read this at the start of each interview)**

*Information about the interview and request for your consent to participate*

- The interview is expected to last approximately 1 hour.
- Your name and any information that can be used to personally identify you will be removed from the data before analysis. Nothing will be directly attributed to you without receiving your permission directly beforehand.
- If something is unclear, please interrupt at any with questions.
- *I* will be taking notes during the interview, and the session is being recorded. Do you agree to recording our conversation?  *[Wait for verbal agreement.]*

**जानकारी आधारित सहमति तथा गोपनीयता कथन (प्रत्येक साक्षात्कार प्रारंभ करने से पहले इसे पढ़ें.)**

*साक्षात्कार की जानकारी तथा भाग लेने हेतु सहमति का अनुरोध*

- साक्षात्कार के लगभग एक घंटा चलने की सम्भावना है.
- आपका नाम अथवा ऐसी कोई भी जानकारी जिससे आपकी पहचान हो सके आंकड़ों का विश्लेषण करने से पूर्व हटा दी जाएगी. बिना आपकी पूर्व अनुमति के किसी भी बात को आपके नाम से नहीं जोड़ा जायेगा.
- यदि किसी प्रश्न में कोई बात स्पष्ट नहीं है तो उसी समय रोककर पूछें.
- मैं साक्षात्कार के दौरान नोट्स लूँगा/गी . इस सत्र की ऑडियो रिकॉर्डिंग भी की जा रही है. क्या आप इस बातचीत की ऑडियो रिकॉर्डिंग के लिए सहमत हैं? *[मौखिक सहमति की प्रतीक्षा करें.]*

The questions we ask you today are related to how your coaching has evolved overtime, benefits of being a coach/coachee, strengths of existing coaching model and recommended refinements to the coaching strategy. There are no right or wrong answers. So, feel free to be as honest as possible. Before we begin, do you have any questions?

आज हम आपसे कोचिंग के विषय में बात करेंगे. समय के साथ इसका कैसे विकास हुआ, कोच अथवा कोची होने के क्या लाभ हैं, अभी अपनाए जा रहे कोचिंग मॉडल की क्या विशेषाएं हैं, कोचिंग रणनीति को और अच्छा बनाने के लिए क्या सुझाव हैं ? इसमें कोई उत्तर सही अथवा गलत नहीं होगा इसलिये बिना किसी संकोच के पूरी ईमानदारी से बात करें. हम अपनी बातचीत आरंभ करें क्या उससे पूर्व आपके मन में कोई प्रश्न है?

**General (Always ask this question first. This is a rapport-building question)**

I would like to begin by asking you a few background questions.

1. Can you tell me more about yourself? In particular, how long have you worked with the city government and how has your work involved family planning?

आप अपने विषय में कुछ बताइए जैसे आप कितने समय से स्वास्थ्य विभाग के साथ काम कर रहे हैं, आपके काम में परिवार नियोजन किस प्रकार शामिल है?

**Coaching history | How you have been coached**

**कोचिंग इतिहास | आपको कैसे कोच किया गया ?**

1. Can you tell me about the training/coaching/technical guidance that you received on urban family planning during your work with government?

- Probe: Please include training/coaching received from government or any other organizations (like who conducted these trainings, what are the content of these trainings, in which year you were trained etc)?

क्या स्वास्थ्य विभाग के साथ काम करते हुए आप को शहरी परिवार नियोजन पर किसी प्रकार का प्रशिक्षण/ कोचिंग/तकनीकी मार्गदर्शन प्राप्त हुआ?

- Probe: कृपया सरकार/अन्य संगठन द्वारा दिए गए प्रशिक्षण/कोचिंग को शामिल करें. (उदहारण के रूप में – ये प्रशिक्षण किसने किए, इनमें क्या सिखाया गया, ये किस वर्ष में हुए आदि)

*IUCD ki as a trainer at the district. I am a trainer for all ANMs particularly for PPIUCD etc.*

1. Can you please describe in detail about the coaching /technical support that you received from TCIHC to strengthen urban family planning services in your city?
   - Probe: Which interventions were you coached on? (for example: utilizing data effectively, planning & budgeting, enabling ASHAs, FDS, Convergence, Mapping & Listing, FTP, etc.)
   - Out of the interventions you listed, which do you find most valuable or useful?

*क्या आप विस्तार पूर्वक बता सकते/ती हैं शहरी परिवार नियोजन को सुदृढ़ करने के लिए आपको TCIHC से क्या कोचिंग/तकनीकी सहयोग प्राप्त हुआ?*

- - Probe: आपको किन HIA पर कोच किया गया? (उदहारण के लिए: आकड़ों का प्रभावशाली उपयोग, योजना तथा बजट बनाना, आशाओं को सक्षम बनाना, नियत सेवा दिवस, कन्वर्जेन्स, मैपिंग तथा लिस्टिंग, एफ.टी.पी. आदि)
  - आपने जिन HIA के विषय में बताया, उनमें से कौन सा आपको सबसे उपयोगी लगा?

9 tools, FDS apki, adolescent 24 UPHCs. 112 Adolescent girls participated in facility AHDs. ASHAs being oriented on how to bring clients. Gap identification, ANM is aware, MAS meeting capacity building

The best one is FDS.

Urban ASHA capacity building

MAS

These are mainly implemented at ground level. IUCD clients day is fixed at UPHC level.

1. When you received this coaching/technical assistance, did your coach use printed job aids/copies of HIAs or refer you to high impact approaches on TCI University or over mobile application. ?
   - Probe: Did you find it easy to use? What resources from TCI U were most useful to you and what could be improved?

**क्या आपकी कोचिंग/प्रशिक्षण के समय आपके कोच ने TCI University पर उपलब्ध TCI के high impact approaches का सन्दर्भ दिया था ? [TCI एप, Infographics (छपे हुए जॉब ऐड) या किसी अन्य माध्यम से]**

- Probe: क्या आपको इन जॉब ऐड का प्रयोग आसान लगा? TCI University पर उपलब्ध कौन सी सन्दर्भ सामग्री सबसे उपयोगी थी? किस सामग्री में और सुधार किया जा सकता है?

Yes, inforgrapics we get attractive hoti hein samajhne mein or samajhane mein hi ahata san hota hein

*I am involved in TCI-U and already registered. There may be some issues, the assessment form is not opening.*

1. How often do you receive coaching/guidance from TCIHC or require an interaction with TCIHC on scaling up HIAs?

- Probe: Is it daily, weekly, monthly, quarterly? Is it intentional (for example: FP review meetings, DHS meeting, CCC meetings, joint visits to a facility) or adhoc where you/they call you? Can you please give some examples?

**आपको TCIHC से प्रायः कितनी बार कोचिंग/ मार्गदर्शन मिलता है? या HIA को क्रियान्वित करने के लिए आपको TCIHC टीम से बातचीत करने की आवश्यकता पड़ती है ?**

**weekly discuss karleete hein. is it structured?**

**if emeregncy then we also call over phone**

**ja jarrorat padti hein ta app. FDS ke din jarror discuss karni hoto hein**

Probe: ये दैनिक, साप्ताहिक, मासिक अथवा त्रैमासिक कैसा होता है? (उदहारण के रूप में: परिवार नियोजन समीक्षा बैठकें, DHS या CCC बैठकें या स्वास्थ्य केन्द्र का साझा भ्रमण ) या ad hoc रूप में होता है कि जब भी आप मिले या उन्होंने आपको फोन किया? क्या आप कुछ उदहारण दे सकते हैं?

**Now I would like to move on to learn more about …**

**How and who you coach** (includes questions on technical coaching)

**अब मैं इस बात को विस्तार से जानना चाहूँगा/गी कि आप किसको और कैसे कोच करते/ती हैं …**

(इसमें तकनीकी कोचिंग के प्रश्न भी शामिल हैं)

1. Who do you coach/train/guide? What is your goal as a coach /trainer within the government system?

**आप किसको कोच/प्रशिक्षित करते हैं अथवा मार्गदर्शन देते हैं ? स्वास्थ्य विभाग में कोच/प्रशिक्षक के रूप में आपका लक्ष्य क्या है?**

Staff nurse, ANM, ASHAs training normally happened at different levels

Lets take an example of IUCD insertion. LT and pharmacist they are also oriented but not like staff nurse

1. On an average, how many coaching/training/guidance sessions do you conduct per month and on what topics and skills?
   - Probe: Of those, how many are scheduled, on demand/ad hoc, or hands on

**एक माह में आप औसतन कितने कोचिंग सत्र करते हैं? और ये किन विषयों और कौशलों पर होते हैं ?**

- - Probe: इनमें से कितने कोची की मांग पर किये गए थे (कोची ने निवेदन किया था) अथवा जब भी आप मिले तब किये गए थे (आप ने आरम्भ किये थे)

4-6 sessions, not only FP. Eligible women, to follow up. Whether we have list lining.

It should cascade properly up to the ground level. FDS. Friday, client. Coaching session so that ASHA should remember the coaching so that she brings clients. Autoclaving, fundamental elementary knowledge

High risk pregnancies, due list by ASHAs, eligible couple line listing. Follow up of the clients for regular method follow up. Origanising/ planning skills ki baat kar rahe hein

1. What proportion of your coaching/training/guidance sessions covers is technical aspect (like how to sterilize instruments, how to indent using FPLMIS) vs. management skills (developing action plans, scheduling review sessions and follow ups) vs. other areas? Has this changed over time?

**आपके कोचिंग/प्रशिक्षण मार्गदर्शन सत्रों में तकनीकी/HIAs, प्रबंधन क्षमता (मैनेजमेंट स्किल), अन्य विषयों का अनुपात क्या होता है? क्या इसमें समय के साथ बदलाव आया है?**

Many changes, technical session was more initially but now it has reversed. Managerial sessions have been increased. Infection control, use of autoclave by staff nurses. If there are more number of clients then we should manage the supply, ensuring supply.

1. Can you share what a typical coaching/training/guidance session looks like for you? (For example: how your session is initiated, how long are your sessions, how are goals established, what do you do with coachee, what job aids you use during your sessions [printed materials, TCI app, TCI U]?).

**क्या आप बता सकते हैं कि एक नियमित कोचिंग सत्र कैसा होता है? (उदहारण के रूप में: आपका सत्र कैसे आरम्भ होता है? आपके सत्र प्रायः कितनी देर चलते हैं? सत्र के लक्ष्य कैसे निर्धारित होते हैं? आप अपने कोची के साथ क्या करते हैं? आप सत्र के दौरान कौन से जॉब ऐड प्रयोग करते हैं - [ छपी हुयी सामग्री, TCI app, TCI U]?).**

MAS meetings,

1. What proportion of your time is spent on coaching /training/guiding coaches versus your other job duties?

- Probe: (depending on the %, follow up with) How is coaching different from your other types of work or other engagements you do with government?

**आपके अन्य कामों में लगने वाले समय और कोचिंग में लगने वाले समय का अनुपात क्या होता है?**

- Probe: (समय के अनुपात के आधार पर फॉलो अप करें) आप जो अन्य काम करते हैं या सरकारी काम के साथ आपका अन्य काम है उसमें और कोचिंग में क्या अंतर है?

Mine is eUPHCs. You are seeing the patients. But at least you can plan the coching when there is a low client volume. 60-70% of the time normally goes on family planning which includes both coaching and other works

**Assess Cascading of Technical coaching (includes questions on technical coaching)**

1. Who does your coachee coach/train/guide?
   - Probe: And do you know what proportion of your coachees time is spent on coaching/training/guiding vs their job duties?
   - Are they mainly coaching on technical aspects or management skills or other?

**आपके कोची किसको कोच करते हैं?**

- - Probe: क्या आपको पता है कि आपके कोची का कितना समय कोचिंग की तुलना में उसके अन्य कामों में लगता है?
  - क्या वे मुख्यतः तकनीकी /HIA बनाम प्रबंधन कौशल पर कोचिंग करते हैं या अन्य किसी विषय पर?

*Staff nurse is my coachee. Check whether it is exactly transferred to ANMs. ANM to ASHA and from ASHAs to MAS members.*

1. What changes have you observed in those you coach/train/guide? (for example, job performance, confidence)

- Probe: Was that knowledge/skill retained over any extended period of time?

**जिन लोगों को आपने कोच किया है उनमें आप क्या परिवर्तन देखते हैं? (उदहारण के रूप में, कार्य प्रदर्शन, आत्मविश्वास)**

- Probe: क्या ये ज्ञान/कौशल लम्बे समय तक बना रहा?

Kayakalp, infection control, any work in OPD related famiy planning, immunization. It increased the confidence of ASHAs to identify the non –users. OPD of family planning is good at 60-70 OPD, 30 clients are on family planning, they come for counselling or receiving services

1. What do you think is needed to build a good relationship between a coach /trainer/guide and coachee/recepient?

- Probe: Have you seen these skills you’ve just outlined being used in those you’ve coached?

**आपके अनुसार कोच एवं कोची के बीच अच्छे संबध बनाने के लिए की किन बातों की आवश्यकता है?**

- Probe: क्या जिन लोगों को आपने कोच किया उनमें ये कौशल हैं?

trust is very important. openness, communication. approach, focus is also important coordination

biswash, sikhna bhi hein or sikhana bhi hein, dono mein apsi taal mel coordination. IUCD lag jaye as an example

1. After receiving technical support/coaching from TCIHC, are you able to implement HIA in the city? What changes have you observed in your city?

**TCIHC से तकनीकी सहायता/कोचिंग मिलने के पश्चात क्या आप शहर में HIA के क्रियान्वयन में सक्षम हैं? आपने अपने शहर में किस प्रकार का परिवर्तन अनुभव किया है?**

People are not aware of the services available at different UPHCs.

We were nit aware of the work at the ground level. ASHA is the main pillar, mapping 3ward muslim population

HRGs client coming from these areas. Yes, at least we have reached to those untouched community. This was possible only after urban ASHAs capacity building

TCIHC ki coaching- bilkul raha hein,

*Tabhi too hame pata chala ki yes ishtarakise kar sakte hein*

This age group 15-19 years is being given services

**Assess Management coaching (includes questions on management coaching)**

1. Can you describe how you coach on effective management? (for example: what component of your coaching is on management vs technical how do they differ with your coachees, if at all…developing action plans, scheduling review sessions and follow ups)

**क्या आप बता सकते हैं कि आप प्रभावी प्रबंधन पर किस प्रकार कोच करते हैं? (उदहारण के रूप में: आपकी कोचिंग का कौन सा भाग प्रबंधन /तकनीकी होता है, क्या वो आपके कोची के तरीके से अलग होता है यदि हाँ तो कैसे ........योजना बनाना, समीक्षा बैठकें आयोजित करना तथा फॉलो अप करना)**

*FDS as an example-due list. ANM will not prepare, this will be prepared by ASHAs.*

**Sustainability and Recommendations for improving coaching**

**निरंतरता तथा कोचिंग को और अच्छा बनाने के लिए सुझाव**

1. Have you seen adoption and adaptation of family planning/AYSRH HIA incorporated into local policies, workplans, guidelines or standards following your coaching? Please provide examples.

**क्या आपने अपनी कोचिंग के बाद FP/ /AYSRH HIA का सरकारी नीतियों, कार्य योजनाओं, दिशा निर्देशों आदि में शामिल किया जाना /अपनाया जाना देखा ? कृपया उदहारण दें.**

*Antaral diwash, kushal pariwar diwash it was happening in rural areas. 21 st every month. These are PMSSY.*

FDS was appreciated a lot, we even did not know whether this will be implemented.

1. How has the government’s ability to take decisions based on data driven decision-making for FP/AYSRH been influenced by TCI coaching? What kind of data review happens?

- Probe: Has TCIHCs technical support assisted you in taking data driven decisions, such as review meetings, prioritizing user/non user

*Clients inflow have increased tremendously.*

- Probe: क्या TCI की तकनीकी सहायता ने आपको आंकड़ों के आधार पर निर्णय लेने में सहायता दी?

Ease of Payments issues, achovmemnt and financial aspects. When FDS started, antara payments is also regularized and now it is in PIP.

1. In your experience, how long does it take to move coachees between different stages – Lead, Assist, Observe?

- Probe: What would you say is a characteristic of a coachee that is ready to take on their own work (ready for Observe stage) that helps prepare them for graduation?
- Probe: Was/Has that knowledge/skill retained over any extended period of time?

**आपके अनुभव में किसी कोची को कोचिंग के तीन चरणों लीड,असिस्ट,ओब्सर्व में एक से दूसरे चरण में पहुँचने में कितना समय लगता है ?**

- Probe: कोई कोची जो अपना काम स्वयं करने को तैयार है, आपके अनुसार इसका महत्वपूर्ण लक्षण क्या है (ओब्सर्व चरण के लिए तैयार) जो उन्हें ग्रेजुएशन के लिए तैयार करता है?
- Probe: क्या यह ज्ञान/ कौशल लम्बे समय तक उस कोची में बना रहा?

1 saal to lagta hein

6 mahine lage the field mein samajhane mein

Mapping etc.

6-12 mahine from assist se observe depending on ASHA level

1. In your opinion, what can be done to improve PSI-TCIHC coaching/technical assistance so that geographies can confidently transition from Lead Assist Observe (Startup, implement/ surge, pre-graduation, and post- graduation)?

**आपके विचार में, TCI कोचिंग में ऐसा क्या सुधार किया जा सकता है जिससे शहरों को विश्वासपूर्वक लीड,असिस्ट,ओब्सर्व में पहुँचाया जा सके (आरम्भ, क्रियान्वयन / सर्ज, प्री-ग्रेजुएशन तथा पोस्ट- ग्रेजुएशन)?**

Your model, this is very unofficial.

1. Have you seen an increase in the number of service providers receiving “observational” coaching for the city’s primary best practice interventions following your coaching/training support?

**क्या आपकी कोचिंग के पश्चात आपके शहर में सबसे पहले प्रारंभ किये गए “बेस्ट प्रैक्टिस” क्रियान्वयन के लिए कोचिंग के ओब्सर्व चरण में सेवा प्रदाताओं की संख्या बढ़ी है?**

**Private nursing homes added in haushala sajhdari program ASHA ko bata diya tha.**

**All 30 UPHCs are activated**

**Transfer and verification =, TCIHC supported HS and that system was developed completely**

1. What successes from your coaching /technical guidance experience would you recommend to other coaches within government (what is your secret sauce that you’ve found most useful) and what should not be done while coaching?

- Probe: What have been challenges you’ve experienced in coaching?
- What lessons would you share with a new coach about “what not to do when coaching within government”?

**अपनी कोचिंगके कौन से सफल अनुभव आप अपने विभाग के अन्य कोच के लिए अनुमोदित करना चाहेंगे? (अपनी सफलता का कौन सा राज़ अप सबसे उपयोगी मानते हैं? और साथ ही ये भी कि कोचिंग के दौरान क्या नहीं करना चाहिए?**

- Probe: आपने अपने कोचिंग अनुभव में किन किन चुनौतियों का सामना किया?
- किसी नए TCI कोच को आप , “ विभाग के लोगो की कोचिंग के दौरान क्या न करें” के बारे में क्या सीख देना चाहेंगे?

9 HIAs as it is iplmented. LAO model to follow, and guideline if yoi=utry to make understand.

The should understand the benefits

Antara vaccine- negative image.

Whether the same thing is getting transferred into lowere level.

They should not be biased

1. Are you aware about RAISE assessments that the government did with the technical support of TCIHC? What role did TCIHC’s coaching/technical assistance /guidance played in it?

- Probe: Do you think coaching is vital to the sustainability of the government conducting RAISE, especially once graduated?

**क्या आप RAISE आंकलन से परिचित हैं जो सरकार द्वारा TCIHC के तकनीकी सहयोग से किया जा रहा है? इसमें TCIHCकोचिंग की क्या भूमिका रही है?**

- Probe: क्या आपको लगता है कि सरकार द्वारा RAISE आंकलन किए जाने की निरंतरता में कोचिंग की भूमिका बहुत महत्वपूर्ण है, विशेष तौर पर जो शहर ग्रेजुएट कर रहे हैं उनके लिए?

Transfer of knowledge and information. Deep analysis, whataever =the gaos comes, we do the action planning. gap analysis

1. Do you have any examples of how coaching/technical assistance has influenced diffusion to non-TCI cities or states? If yes, how do you think this happened?

**क्या आपके पास ऐसा कोई उदहारण है जिसमें कोचिंग ने नॉन- TCI शहरों या राज्यों में विसरण (डिफ्यूजन) को प्रभावित किया हो ? यदि हाँ, तो आपको क्या लगता है ये कैसे हुआ होगा ?**

There are two things, if we have to tell aout anataral diwas,

1. Self-Reflection: What will TCI leave behind in the city after all of this coaching/technical assistance?

- Probe: How do you feel coaching has been institutionalized?
- What can be improved in the level of coaching from TCIHC?

**स्व-आंकलन: इस पूरी कोचिंग प्रक्रिया के परिणाम स्वरुप TCI शहर में क्या छोड़कर जायेगा?**

- Probe: क्या आपको लगता है कि कोचिंग संस्थागत हो गयी है?
- TCIHC के स्तर पर कोचिंग में और क्या सुधार किया जा सकता है?

We have reached to positivity, mind set changed.

HIAs

Thank you very much for participating in this interview. We appreciate that you have shared your valuable expertise, perspectives, and time with us. We hope that we can contact you again if we need to clarify any questions.

**इस साक्षात्कार में भाग लेने के लिए धन्यवाद. आपने समय निकालकरअपने मूल्यवान विचार और अनुभव हमारे साथ साझा किए इस हेतु हार्दिक आभार. यदि किसी प्रश्न पर और अधिक स्पष्टता की आवश्यकता हुयी तो हम आपसे पुनः संपर्क करेंगे.**

आशा समूह चर्चा हेतु प्रस्तावित दिशानिर्देश

(Coaching Study)

| समूह चर्चा सुगमकर्ता का नाम (Name of the Facilitator) |  | | | |
| --- | --- | --- | --- | --- |
| नोट्स लेने वाले का नाम |  | | | |
| दिन, दिनांक तथा समय |  | | | |
| स्थान |  | | | |
| प्रतिभागियों का परिचय | क्रम | नाम | स्वास्थ्य केंद्र का नाम | अनुभव (वर्ष) |
|  | 1 |  |  |  |
|  | 2 |  |  |  |
|  | 3 |  |  |  |
|  | 4 |  |  |  |
|  | 5 |  |  |  |
|  | 6 |  |  |  |

नमस्कार. मेरा नाम ...................है और मैं TCIHC टीम की सदस्य हूँ. सबसे पहले आप सभी को आज की समूह चर्चा में प्रतिभाग करने के लिए आने हेतु बहुत बहुत धन्यवाद. आज हम आपसे कोचिंग के विषय में बात करेंगे. आप सभी ने एफ.पी.ए. दीदी के साथ किया है. वो आपको सारे काम पहले कर के दिखाती थीं, फिर आपसे करने को कहती थीं और बीच बीच में आवश्यकता के अनुसार आपको सहायता देती थीं, फिर वो काम आप करने लगती थीं और एफ.पी.ए. दीदी आपको देखती थीं और फीडबैक देती थीं. यही कोचिंग का लीड (करके दिखाना), असिस्ट (करने में सहायता देना), ओब्सर्व (करते हुए देखना) यानि LAO मॉडल है. हम जानना चाहते हैं कि इस पद्धति से सीखते हुए आपको कैसा लगा? क्या इसमें सीखते समय आपके सामने कुछ चुनौतियाँ आयीं? या क्या आपके पास इसको और अच्छा बनाने के कुछ सुझाव हैं?

इस समूह चर्चा का उद्देश्य उन बातों का पता लगाना है जिससे कोचिंग की इस पद्धति को और अच्छा बनाया जा सके. इस तरह की समूह चर्चा हम तीन प्रदेशों में पांच शहरों की आशाओं के साथ कर रहे हैं.

हमारी ये समूह चर्चा लगभग एक-से डेढ़ घंटे चलेगी. इस समूह चर्चा में आप जो कुछ भी कहेंगी उसका उपयोग केवल अध्ययन के उद्देश्य से किया जायेगा. आपका नाम कहीं भी सामने नहीं आएगा पूरी तरह गोपनीय रखा जायेगा. इस समूह चर्चा में पूछे गए प्रश्नों के कोई भी सही या गलत उत्तर नहीं होंगे इसलिए जो भी आप सोचती हैं खुले मन से हमारे साथ साझा करें.

समूह चर्चा प्रारंभ करने से पहले संक्षेप में परिचय कर लेते हैं. आप सब बारी बारी से परिचय दीजिये – अपना नाम, अपने स्वास्थ्य केंद्र का नाम, आशा के रूपमे कितने वर्षों का अनुभव है.

**Coaching History and Coaching Experience**

1. शहरी आशा के रूप में आप क्या क्या काम करती हैं? स्वास्थ्य के कौन कौन से मुद्दों पर आपको काम करना होता है?
2. आपके काम में लाभार्थियों को परिवार नियोजन सेवाओं से जोड़ना किस प्रकार शामिल है? आप परिवार नियोजन सेवाओं से जुड़े क्या क्या काम करती हैं?
3. क्या स्वास्थ्य विभाग के साथ काम करते हुए आप को शहरी परिवार नियोजन पर किसी प्रकार का प्रशिक्षण/कोचिंग/तकनीकी मार्गदर्शन प्राप्त हुआ? (Probe: कृपया सरकार/अन्य संगठन द्वारा दिए गए प्रशिक्षण/कोचिंग को शामिल करें. उदहारण के रूप में – ये प्रशिक्षण किसने किए, इनमें क्या सिखाया गया, ये किस वर्ष में हुए आदि)
4. आपको PSI/TCIHC के द्वारा किस प्रकार प्रशिक्षण तथा कोचिंग प्रदान की गयी? आपको TCIHC से प्रायः कितनी बार कोचिंग/ मार्गदर्शन मिलता है? या HIA को क्रियान्वित करने के लिए आपको TCIHC टीम से बातचीत करने की आवश्यकता पड़ती है ?
5. TCIHC से आने वाली एफ.पी.ए. दीदी ने आपको किस किस विषय पर कोचिंग दी? (प्रोब, जैसे - गृह भ्रमण कैसे करें? गृह भ्रमण के दौरान क्या करें? परिवार नियोजन लाभार्थियों की प्राथमिकता सूची कैसे बनायें? UHIR updation तथा अन्य).
6. क्या इस कोचिंग के दौरान एफ.पी.ए. दीदी ने किसी छपी हुयी सामग्री को दिखाया / फोन पर दिखाया ?(When you received this coaching, did FPA use printed job aids/copies of HIAs or refer you to high impact approaches on TCI University or over mobile application?
7. क्या TCIHC से आपको कुछ टूल्स ( गृह भ्रमण/ परामर्श के दौरान उपयोग की सामग्री) भी प्राप्त हुए हैं? ये कौन से टूल हैं, आप इनका प्रयोग कब और कैसे करती हैं? (प्रोब: बास्केट ऑफ़ चॉइस, परिवार नियोजन परामर्श पुस्तिका आदि)
8. TCIHC से आने वाली एफ.पी.ए. दीदी की कोचिंग करने की पद्धति क्या थी? (वो आपको कोई भी चीज़ कैसे सिखाती थीं? ये पद्धति आपको कैसी लगी? (उनका सिखाने का तरीका आपको कैसा लगा? (tell us in detail)
9. क्या TCIHC के आने के बाद आपके प्राथमिक स्वास्थ्य केंद्र पर कुछ नयी गतिविधियाँ प्रारंभ हुयी हैं ? ये गतिविधियाँ कौन सी हैं?
10. क्या एफ.पी.ए. दीदी ने आपको अन्तराल दिवस के विषय में भी विस्तार से सिखाया था ? क्या सिखाया था? अंतराल दिवस में आपकी क्या भूमिका है?
11. आप कैसे अनुमान लगा लेती हैं कि किस क्लाइंट को परिवार नियोजन की सबसे ज्यादा आवश्यकता है?
12. क्या आप अपने क्षेत्र का 2BY2 मैट्रिक्स प्रतिमाह बनाती हैं? कैसे?
13. मान लीजिये कि मैं एक नयी आशा हूँ और अपनी डायरी लेकर आपके पास आई हूँ तो क्या आप मेरे क्षेत्र की भी ड्यू लिस्ट/ 2BY2 बनवा सकती हैं? कैसे?
14. एफ.पी.ए. दीदी के जाने के बाद से आपको अपने विभाग से कौन कौन कोचिंग देता है? ये लोग आपको किस किस विषय पर कोचिंग देते हैं? (Probe: UHIR updation, 2By2 matrix, what are the platforms where ANMs coach ASHAs (weekly meetings, monthly meetings at UPHCs, how FPCs are assisting/observig their ANMs while ANM is coaching ASHAs, coaching conducted by higher city officials on indenting logistics etc)
15. आप व्यक्तिगत रूप से परिवार नियोजन कार्यक्रम को कितना महत्वपूर्ण मानती हैं? (क्या TCIHC की कोचिंग से पहले भी आप परिवार नियोजन को इतना ही महत्वपूर्ण मानती थीं?
16. TCIHC से कोचिंग प्राप्ति के पश्चात् आप क्षेत्र कार्य/ परामर्श आदि में स्वयं को कितना सक्षम मानती है? अथवा क्या कोचिंग से आपकी क्षमताओं में कुछ वृद्धि हुयी है? TCIHC के साथ काम करके आपने ऐसा क्या सीखा जो आपको लगता है अब सदा आपके साथ रहेगा?
17. क्या कोचिंग मिलने के बाद आप अपना काम बेहतर रूप से कर पा रही है? (or would you require more support? probe: what specific support would you require?)
18. क्या पिछले वर्षों में आपने TCIHC के कारण स्वास्थ्य केंद्र/समुदाय स्तर पर इस कार्यक्रम के पहले की तुलना में किसी प्रकार का परिवर्तन देखा है? (What is different in the facility/community now because of TCIHC, compared to when we started? What hasn’t changed? What areas need more attention or focus?)
19. क्या आपके मन में TCIHC कोचिंग को बेहतर बनाने के लिए कोई सुझाव है?
